# Single‐nucleus RNA sequencing reveals the distinct heterogeneity of primary pulmonary sarcomas (PPS) and pulmonary sarcomatoid carcinoma (PSC)

**DOI:** 10.1002/ctm2.70566

**Published:** 2025-12-30

**Authors:** Jianfei Zhu, Yanlu Xiong, Shouzheng Ma, Jun Wei, Jiakuan Chen, Wenchen Wang, Qianqian Duan, Qin Zhang, Dongsheng Chen, Wanglong Deng, Tao Jiang, Jie Lei

**Affiliations:** ^1^ Department of Thoracic Surgery The Second Affiliated Hospital, Air Force Medical University Xi'an China; ^2^ Department of Thoracic Surgery Air Force 986th Hospital, Air Force Medical University Xi'an China; ^3^ The State Key Laboratory of Neurology and Oncology Drug Development Jiangsu Simcere Diagnostics Co., Ltd Nanjing China; ^4^ Department of Medicine Nanjing Simcere Medical Laboratory Science Co., Ltd. Nanjing China

**Keywords:** primary pulmonary sarcomas, pulmonary sarcomatoid carcinoma, single‐nucleus RNA sequencing, TME

## Abstract

**Background:**

Primary pulmonary sarcomas (PPS) and pulmonary sarcomatoid carcinoma (PSC) are rare and aggressive diseases that pose significant diagnostic challenges, requiring extensive sampling and comprehensive evaluation. To date, the single‐cell characteristics and distinctions between these two conditions have not been thoroughly investigated.

**Methods:**

In this study, we employed single‐nucleus RNA sequencing (snRNA‐seq) to characterise the cellular heterogeneity of PSC and PPS. Our analysis included 20 PSC samples, seven PPS samples and two non‐malignant control samples obtained from adjacent normal tissue.

**Results:**

Our results revealed that the majority of cells in PSC were of epithelial origin, while fibroblasts predominated in PPS. Specifically, AT2 cells, a major source of epithelial cells in PSC, underwent malignant transformation primarily through epithelial–mesenchymal transition, suggesting AT2 cells may serve as the origin of PSC. High Mobility Group AT‐Hook 2 (HMGA2) expression was elevated in malignant AT2 cells of PSC and correlated with an unfavourable prognosis. Moreover, MET‐mutated patients have a significantly higher expression level of HMGA2 (*p* < .001). In PPS, fibroblasts constituted the majority, only lipofibroblasts exhibited malignant features. A direct comparison between PSC and PPS lipofibroblasts revealed largely similar expression profiles, with the exception of an enrichment in DNA repair pathways specifically observed in PPS lipofibroblasts.

**Conclusion:**

These findings provide novel insights of PSC and PPS at the single‐cell level.

**Key points:**

- **Distinct Cellular Origins**: PSC arises primarily from epithelial (AT2) cells via EMT, whereas PPS is predominantly fibroblast‐derived.
- **Prognostic Driver HMGA2**: Elevated HMGA2 in malignant AT2 cells correlates with poor prognosis and is significantly higher in MET‐mutated PSC.
- **Pathway Divergence in Malignant Cells**: Malignant lipofibroblasts in PPS share a similar profile with PSC but uniquely enrich DNA repair pathways.

## Graphical abstract

Single‐nucleus RNA sequencing (snRNA‐seq) maps the cellular diversity of pulmonary sarcomatoid carcinoma (PSC) and primary pulmonary sarcomas (PPS). AT2 cells, a major source of epithelial cells in PSC, underwent malignant transformation primarily through epithelial–mesenchymal transition, suggesting AT2 cells may serve as the origin of PSC. HMGA2 expression was elevated in malignant AT2 cells of PSC and correlated with an unfavourable prognosis. In PPS, fibroblasts constituted the majority, lipofibroblasts exhibited malignant features. Comparison between PSC and PPS lipofibroblasts revealed largely similar expression profiles, with the exception of an enrichment in DNA repair pathways specifically observed in PPS lipofibroblasts.

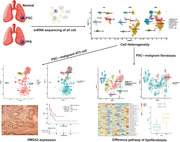

## 1 BACKGROUND

Pulmonary sarcomatoid carcinoma (PSC) and primary pulmonary sarcomas (PPS) both represent infrequent and highly aggressive neoplasms of the lung. The biphasic lung carcinoma known as PSC, characterised by a combination of epithelial and sarcomatous components, is an exceedingly rare entity, accounting for only .1%–.4% of all primary lung neoplasms.^1^ PSC subtypes exhibit a range of deviations from the typical epithelioid morphology seen in non—small‐cell lung cancer (NSCLC). These neoplasms may contain spindle‐shaped or giant cells that resemble sarcomas, as well as heterologous sarcomatous components or rhabdomyosarcomatous differentiation. The WHO classifies tumours as PSC when these morphological transformations are present in at least 10% of the neoplasm.^2^, ^3^, ^4^ While PPS which was raised from mesenchymal tissue of the bronchial wall vessels or pulmonary stroma that are not a metastasis from an extrathoracic sarcoma are reported to constitute only .1%–.3% of all lung malignancies.^5^ The frequently observed histological subtypes include synovial sarcoma, epithelioid hemangioendothelioma, leiomyosarcoma and malignant peripheral nerve sheath tumour. Subsequently, undifferentiated pleomorphic sarcoma, liposarcoma and rhabdomyosarcoma are also commonly encountered. At present, pathophysiological mechanisms and molecular pathways underlying PPS are largely unknown.^6^ The clinical and imaging characteristics of PPS and PCS resemble those observed in other subtypes of NSCLC. Extensive tissue sampling is the key to distinguishing PCS from PPS.^7^ In addition, they frequently manifest with regional invasion and distant metastasis. Owing to their more aggressive nature, both PPS and PCS exhibit a relatively unfavourable prognosis compared to other forms of NSCLC.^8^ According to several studies, the poor prognosis of both tumour types can be attributed to the aggressive behaviour of their sarcomatous components.^7^, ^9^ Due to the non‐specific symptoms, the rarity of the disease, and the intratumoural heterogeneity (ITH) makes the diagnosis and treatment of PSC and PPS particularly tricky.^10^ Considering the infrequency of PSC and PPS cases, conducting a randomised controlled trial with sufficient participant numbers becomes an arduous task in determining the most effective treatment strategy for both PSC and PPS. The involvement of thoracic surgeons within lung cancer multidisciplinary teams is seldom sought when making decisions related to managing patients with PSC. This circumstance might contribute to a pessimistic outlook concerning treatment options and prognostic outcomes.

Recently, the ITH of PSC have presented by the multi‐omics analysis including whole‐exome sequencing (WES), RNA sequencing test and methylation sequencing demonstrated molecular characteristics and relationship with targeted therapy and immunotherapy.^11^, ^12^ While, regards to PPS, existing literature regarding PPS has been limited only to small retrospective case series that have examined the impact of tumour pathology and clinical management strategies on overall survival (OS).^6^ The similarities and differences between PSC and PPS at the single‐cell level have not been studied so far. Consequently, a comprehensive exploration of the molecular characteristics of PSC and PPS, particularly at the single‐cell level, can assist us in comprehending their molecular mechanisms and also offer some guidance for the management of postoperative treatment.

In this study, we conducted single‐nucleus RNA sequencing (snRNA‐seq) analysis on PSC, PPS and normal control lung tissues. We characterised the interaction profiles of tumour cells and tumour microenvironment (TME) cells in PSC and PPS. Our data provide an in‐depth understanding of PSC and PPS, and may provide a resource for the accurate diagnosis and treatment.

## 2 METHODS

### 2.1 Patients and sample

All participants were enrolled from January 2015 to December 2020; the last follow‐up was in December 2022. Inclusion criteria: (1) All patients with PSC and PPS were those who had undergone surgical; (2) Informed, written consents were obtained from all participants prior to specimen collection; (3) Sample of surgery diagnosed by immunohistochemistry (IHC) combined with haematoxylin–eosin (H&E) staining. All tumour specimens were reviewed by at least two independent pathologists according to the WHO (5th edition) lung tumour or soft tissue and bone tumours classification. For PSC, elements of both NSCC (usually squamous cell carcinoma or adenocarcinoma) and sarcoma with heterologous elements (rhabdomyosarcoma, chondrosarcoma and osteosarcoma) must be present. The IHC of the carcinomatous element corresponds to that seen in typical tumours of the same subtype: TTF1 and napsin A are expressed in adenocarcinoma, while p40 is positive in squamous cell carcinoma.^2^, ^13^ For heterologous sarcomatous elements, markers such as desmin or myogenin can identify rhabdomyosarcomatous differentiation, whereas S100 is indicative of chondrosarcomatous components. Extensive tissue sampling and sarcomatous element marker are used to identify PPS. Exclusion criteria: (1) Patients with a history of malignant tumours should be excluded; (2) Exclude patients who have received chemotherapy and radiation therapy before surgery; (3) Patients with insufficient samples or substandard sample quality should be excluded during the experiment. This study involving human participants were reviewed and approved by the Ethics Committee (TDLL‐202204‐01). We collected a total of 51 samples. After the cell nuclei isolation from cell suspensions, 21 samples ultimately met the quality control (QC) standards and were sequenced, including twenty PSC, seven PPS and two non‐malignant samples (normal control from adjacent tumour).

### 2.2 Haematoxylin–eosin staining and immunohistochemistry

PSC and PPS must be diagnosed by a combination of H&E and IHC from surgically resected specimens. The diagnosis of PSC must meet the following two conditions: CK is positive in epithelial cells and Vimentin was positive in spindle cells and negative in epithelioid cells. The diagnosis of PPS should be consistent with the fact that CK is negative and Vimentin is positive in spindle cells. All diagnoses were made by three independent experienced pathologists according to the WHO (5th Edition) criteria for classification (online version) of thoracic tumours and soft tissue and Bone tumours.^14^

High Mobility Group AT‐Hook 2 (HMGA2) expression was performed using HMGA2 (20795‐1‐AP, 1:500, Proteintech) monoclonal antibody using an automatic immunostainer and Ultra VISION universal DAB (3,3′‐diaminobenzidine) detection kit (Ventana Medical Systems, Inc.). Appendix tissue sections were used as the positive and negative controls. All microscopic analyses were performed using an optical microscope (Zeiss).

### 2.3 Cell preparation—Nuclei isolation from cell suspensions

After harvested, tissues were dissociated using Isolation of Nuclei for Single Cell RNA Sequencing (10× Genomics Catalog No. CG000124) reference 10× Genomics guideline and literatures as instructions.^15^, ^16^ Nuclei count and viability were estimated using fluorescence Cell Analyzer (Countstar® Rigel S2) with acridine orange/propidium iodide reagent after removal debris. Finally, nuclei were washed twice in the 1 × phosphate buffered saline (PBS) and 1% bovine serum albumin (BSA) and .2U/µL RNase Inhibitor and then resuspended at 1 × 10^6^ nuclei per mL in 1 × PBS and 1% BSA and .2 U/µL RNase Inhibitor BSA.

### 2.4 snRNA‐seq library construction and sequencing

Single‐cell RNA‐Seq libraries were generated using the Chromium Next GEM Single Cell 3ʹ Reagent Kits v3.1 (10× Genomics Catalog No. 1000121). In summary, a suitable quantity of cells was combined with reverse transcription reagents and introduced into the sample well of the Chromium Next GEM Chip G. Gel beads and partitioning oil were then added to the designated wells of the chip. Following the formation of emulsion droplets, reverse transcription was carried out at 53°C for 45 min, followed by enzyme inactivation at 85°C for 5 min. The resulting cDNA was then extracted from the broken droplets and subjected to amplification via polymerase chain reaction (PCR). After amplification, the cDNA products were purified, fragmented, end‐repaired, A‐tailed, and connected to sequencing adapters. Afterwards, indexed PCR was conducted to enrich DNA fragments corresponding to the 3′ polyA regions of expressed genes, which also included cell barcodes and unique molecular identifiers (UMIs). The final indexed libraries were purified using SPRI beads, quantified using qPCR (KAPA Biosystems KK4824) and sequenced on an Illumina NovaSeq 6000 platform with a paired‐end read length of 150 bases.

### 2.5 snRNA‐seq data quality control and integration

**EXTENDED DATA FIGURE 1:**
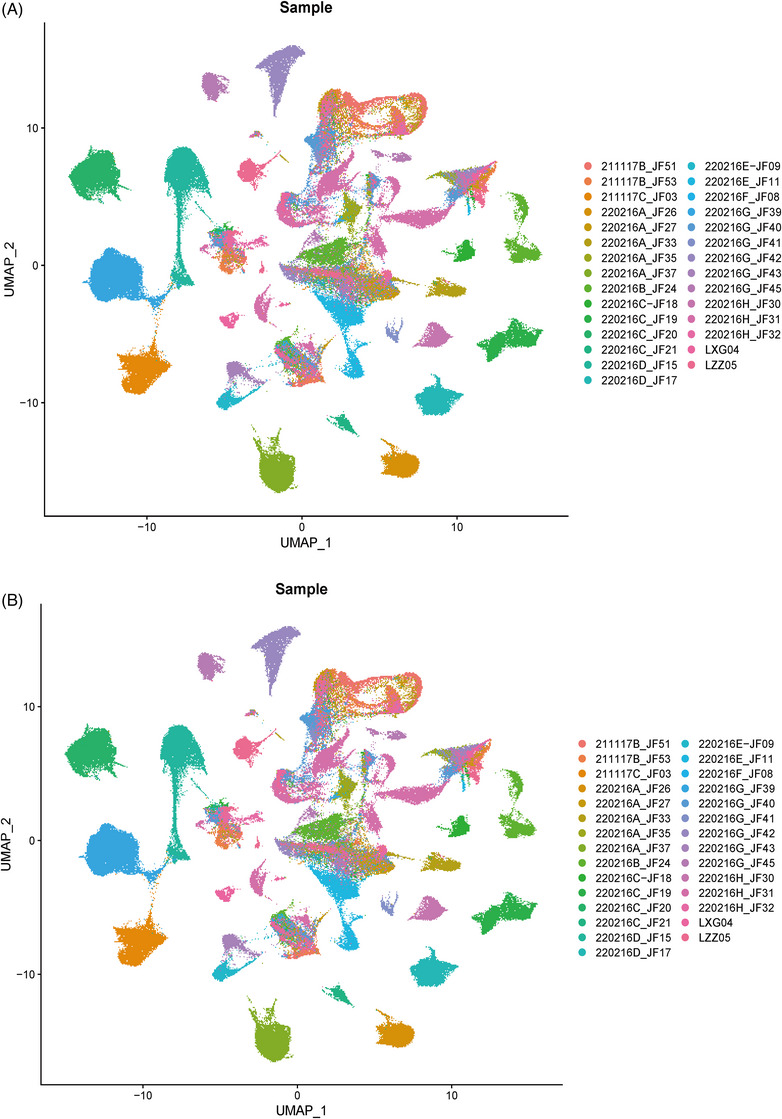
The quality control results of all samples. (A) UMAP of all samples before batch correction. (B) The results after batch correction with RunHarmony

The raw gene expression matrices generated using CellRanger (version 6.1.2) were integrated in R (version 4.1.1) and converted to a Seurat object using the Seurat package (version 4.0.5).^17^ For each cell, we used four QC measures. Cells meeting any of the following criteria were excluded: (1) fewer than 700 expressed genes, (2) >20% UMIs of mitochondria genes, (3) (UMI) count <1000, (4) the number of genes detected per UMI at log scale <.8; After applying the filtering criteria, the final dataset comprised from a total of 181 764 cells. Subsequently, integrated analyses were conducted using Seurat. The filtered gene expression matrix was initially normalised using the SC Transform method^18^ based on the top 3000 highly variable features calculated by vst method. Subsequently, batch effects were mitigated utilising the Harmony package (version 1.0). Ultimately, a batch‐corrected expression matrix encompassing all cells was obtained for subsequent downstream analysis. The results before and after batch correction are shown in Extended Data Figure 1.

### 2.6 Cell clustering and annotation

The clustering analysis was performed using the integrated joint embedding generated by Harmony, followed by application of the Louvain algorithm to compute a shared nearest‐neighbour graph. This graph was then utilised within the ‘FindClusters’ function of the Seurat package. The resolution was set to .8 to obtain the clustering results. The identified clusters were visualised on the two‐dimensional (2D) map produced with the uniform manifold approximation and projection (UMAP) method. Cell clusters were annotated by identifying differentially expressed genes (DEGs) with strong discriminatory abilities between the groups using the FindAllMarkers function in Seurat, employing the default non‐parametric Wilcoxon rank sum test with Bonferroni correction. The cell groups were annotated based on the DEGs and the well‐known cellular markers (Extended Data Table 1). Subsequently, we used SingleR^19^ to make annotations for each cell type. SingleR identified cell types based on similarities in expression patterns between the cells to be identified and the reference cells.

**EXTENDED DATA TABLE 1:** Marker genes in different cell types.

Cell type	Markers
Club cell	SCGB3A2, CCKAR
Ciliated cell	FOXJ1, TUBB1, TP73, CCDC78
Basal cell	KRT5, KRT14, TP63, DAPL1
Goblet cell	MUC5B, MUC5AC, SPDEF
Mucous cell	MUC5B
Serous cell	PRR4, LPO, LTF
Ionocyte cell	CFTR, FOXI1, ASCL3
Neutrophil	CALCA, CHGA, ASCL1
Brush cell (Tuft cell)	DCLK1, ASCL2
Alveolar cell type 1	AGER, PDPN, CLIC5
Alveolar cell type 2	SFTPB, SFTPC, SFTPD, MUC1, ETV5
Arterial cell	GJA5, BMX
Vein cell	ACKR1
Capillary cell	CA4
Lymphatic endothelial cell	PROX1, PDPN
Vascular smooth muscle cell	CNN1, ACTA2, TAGLN, RGS5
Airway smooth muscle cell	CNN1, ACTA2, TAGLN, DES, LGR6
Fibroblast	COL1A1, PDGFRA
Myofibroblast	COL1A1, PDGFRA, ELN, ACTA2
Lipofibroblast	COL1A1, PDGFRA, PLIN2, APOE
Pericyte	CSPG4, TRPC6, PDGFRB
Mesothelial cell	MSLN, UPK3B, WT1
Intrinsic neuron	SNAP25
B cell	CD79A, CD24, MS4A1, CD19
Plasma cell	CD79A, CD27, SLAMF7
Effector CD8+ memory T (Tem) cell	CD3E, CD8A, GZMK, DUSP2
Naive CD8+ T cell	CD3E, CD8A, GZMH, GZMB
Effector CD4+ memory T (Tem) cell	CD3E, CD8A, COTL1, LDHB
Naive CD4+ T cell	CD3E, CD4, CCR7, LEF1
Natural killer T (NKT) cell	CD3E, CD8A, FCER1G, TYROBP
Natural killer cell	KLRD1, NKG7, TYROBP
Neutrophil	S100A8, S100A9, IFITM2, FCGR3B
Basophil	MS4A2, CPA3, TPSAB1
Mast cell	MS4A2, CPA3, TPSAB1
Eosinophil	SIGLEC8
Megakaryocyte	NRGN, PPBP, PF4, OST4
Macrophage	MARCO, MSR1, MRC1
Plasmacytoid dendritic cell (PDcs)	LILRB4, IRF8, LILRA4
Monocyte	CD14, S100A8
Intermediate monocyte	CD14, S100A8, CD16
Non‐classical monocyte	CD16
T cell	CD3, CD4, CD8, CD25
CD4+ T cell	CD4
CD8+ T cell	CD8A, CD8B
Naive CD4+ T cell	CCR7, LEF1, SELL, TCF7
Regulatory T (Treg) cell	IL2RA, FOXP3, IKZF2
Effector T cell	GNLY, CX3CR1, PRF1, GZMA, GZMB
Naive CD8+ T cell	CCR7, LEF1, SELL, TCF7
Exhausted CD8+ T cell	LAYN, ITGAE, PDCD1, CTLA4, HAVCR2, LAG3, TIGIT
B cell	CD79A, IGKC
Follicular B cell	MS4A1
Marginal zone B cell	JCHAIN
Plasma cell	IGHG1
CD4+ T cell	CD4
Regulatory T (Treg) cell	IL2RA
Natural killer cell	FGFBP2
T cell	CD3D, CD3E
Myeloid cell	LYZ, CD68
Macrophage	CD163
Granulocyte	S100A12
Langerhans cell	FCER1A
Monocyte derived dendritic cell	CLEC9A, DUSP4
Fibroblast	DCN, C1R
Endothelial cell	PECAM1, RAMP2
Mast cell	TPSB2, CPA3
Cancer cell	EPCAM, SOX4
Alveolar cell	SFTPC, SFTPA1
Epithelial cell	CAPS, TPPP3
Arterial cell	GJA5, IL33, SOX17
Dendritic cell	HSPG2, VWA1, ENG
Endothelial tip cell	PGF, CXCR4, LXN
Alveolar cell type 1	HPGD, IL1RL1, EDNRB
Alveolar cell type 2	FCN3, BTNL9, NOSTRIN
Intermediate cell	SFTPC
Oligodendrocyte	OLIG1, OLIG2, MOG, CLDN11
Mast cell	KIT, MS4A2, GATA2
Myeloid cell	LYZ, MARCO, CD68, FCGR3A
B cell	CD79A, IGHM, IGHG3, IGHA2
Natural killer cell	NKG7, GNLY, NCAM1, KLRD1
T cell	CD3D, CD3E, CD3G, TRAC
Endothelial cell	PECAM1, CLDN5, FLT1, RAMP2
Fibroblast	DCN, THY1, COL1A1, COL1A2
Epithelial cell	EPCAM, KRT19, KRT18, CDH1
Effector T cell	CD8A
Regulatory T (Treg) cell	FOXP3
T helper cell	CD4
Alveolar cell	CLDN18
Endothelial cell	CLDN5
Epithelial cell	CAPS
Fibroblast	COL1A1
B cell	CD79A
Myeloid cell	LYZ
T cell	CD3D
Cancer cell	EPCAM
T cell	CD3D, CD3G, CD3E, CD2
Natural killer cell	KLRF1, KLRB1, KLRD1, FGFBP2
Myeloid cell	AIF1, LYZ, CD68, MS4A7
B cell	MS4A1, CD79A, CD79B, BANK1
Plasma cell	JCHAIN, MZB1, IGHG1, IGHG2
Mast cell	TPSB2, TPSAB1, CPA3, MS4A2
Fibroblast	ACTA2, DCN, COL1A2, COL1A1
Endothelial cell	CLDN5, VWF, RAMP2, CCL21
Epithelial cell	EPCAM, TPPP3, AGER, SFTPA1
Erythroblast	HBM, HBA1, HBA2, HBD
Alveolar cell type 1	AGER, CAV1, EMP2
Alveolar cell type 2	SFTPA1, SFTPA2, SFTPC, PGC
Club cell	SCGB1A1, TSPAN8, BPIFB1, TMEM45A
Basal epithelial cell	KRT17, KRT6A, KRT5
Ciliated cell	TPPP3, CAPS, TMEM190, C1orf194
Macrophage	APOE
Monocyte	FCN1
Monocyte derived dendritic cell	MRC1, CD14
Plasmacytoid dendritic cell (PDcs)	LILRA4
Granulocyte	G0S2
Alveolar macrophage	PPARG, APOE
Perivascular macrophage	LYVE1, APOE
Inflammatory macrophage	CHI3L1, TNF, AXL
Tumour‐associated macrophage	VEGFA, APOE
Classical monocyte	CD14, FCN1
Non‐classical monocyte	FCGR3A, FCN1
Conventional dendritic cell I	CLEC9A
Conventional dendritic cell II	CD1C, CD207
Myofibroblast	ACTA2, PTN

### 2.7 Copy number variation estimation and identification of malignant cells

Malignant cells were identified using infer copy number variation (CNV).^20^, ^21^, ^22^ InferCNV which works by finding cells with large copy number variations as determined by sorting expressed genes by their chromosomal location and applying a moving average, a sliding window of 100 genes within each chromosome, to the relative expression values. All cells from two normal samples were used as reference controls. We scored each cell for the extent of CNV signal and plotted cells on a dendrogram which was then cut at the highest point in which all the spiked in control cells belonged to one cluster All cells that clustered together with spiked in controls were labelled ‘non‐malignant’, whereas the remaining two clusters were labelled as ‘malignant’.

### 2.8 Functional enrichment analysis and gene set variation analysis

The R package ClusterProfiler 4.0.5^23^ was used for gene ontology (GO), Kyoto encyclopaedia of genes and genomes (KEGG) and gene set enrichment analysis (GSEA) analysis of the differential marker genes among subclusters. To assign pathway activity estimates to individual cell type or sample, we applied gene set variation analysis (GSVA) using standard settings, as implemented in the GSVA R package (version 1.40.1), as described previously.^24^ The gene set of 50 hallmark pathways we investigated (h.all.v7.2.symbols.gmt) was downloaded from the GSEA website (https://www.gsea‐msigdb.org/gsea/index.jsp).

### 2.9 Epithelial and fibroblasts subset analysis

Cells previously annotated as epithelial (*n* = 41 648) and fibroblasts (*n* = 89 622) were subset and reclustered using methods described above and the following parameters: Ngenes = (2000), Npcs = 20, Res = .7, Kparam = 30. Gene signatures were compiled using differential expressed as well as known cell marker genes. The remaining signatures were identified directly from top DEGs.

### 2.10 Trajectory analysis

#### 2.10.1 RNA velocity

The sorted bam files were used to generate loom files using the velocyto run10× function with the genome annotation file ‘GRCh38.gtf’. The loom files for each sample were merged into one loom file. velocyto.R package (version 0.6) was used to calculate the velocity and visualise on plot, we used the method using steady‐state mode, following the Seurat to RNA velocity guides (https://github. com/basilkhuder/Seurat‐to‐RNA‐Velocity).

#### 2.10.2 Monocle2

We used Monocle2 (version 2.14.0) to infer the cell lineage trajectory of AT2 cells with the top 1000 signature genes with *q* value <.001 calculated by differential GeneTest function. The differentiation trajectory was inferred with the default parameters of Monocle after dimension reduction and cell ordering.

### 2.11 Simultaneous gene regulatory network analysis

SCENIC30 is an innovative computational approach designed for building regulatory networks and discerning distinct cellular states using scRNA/snRNA‐seq datasets. To assess variations among cell clusters in terms of transcription factors or their associated target genes, SCENIC was applied across all individual cells, and the Limma package was utilised to determine the regulons with preferential expression.^25^ Only those regulons exhibiting significant upregulation or downregulation in at least one cluster, with an adjusted *p* value below .05, were included in subsequent analyses.

### 2.12 Cell–cell communication analysis

We evaluated disparities in putative cell–cell communication modules among different cell types within each group (PSC and PPS) by integrating the gene expression data using CellChat (version 1.1.3).^26^ Following the standard CellChat pipeline, we utilised the default CellChatDB as the ligand–receptor database and subsequently inferred cell type‐specific communication by identifying upregulated ligands or receptors in one cell group, followed by identification of augmented ligand–receptor interactions upon overexpression of either the ligand or receptor. We then used NicheNet to investigate the signalling mediators involved in ligand–receptor pairing among cancer cells signalling with immune cells (T cells, B cells, macrophages), epithelial cells and fibroblasts. During the NicheNet runs, we evaluated the interaction between ligands and receptors. For both the sender and receiver populations, downstream analysis was conducted using genes from the signalling pathway of interest that exhibited expression in at least 10% of the cells. NicheNet analysis was conducted utilising the vignette to rank potential ligands, infer receptors and predict top target genes of the identified ligands.

### 2.13 Genomic profile analysis

For the remaining tissue samples (17 PSC and seven PPS), we conducted further DNA‐level mutation analysis (detecting 539 tumour‐related genes). Genomic DNA (gDNA) was isolated using a DNA extraction kit designed for tissue samples (Kai Shuo). The indexed paired‐end adapters compatible with the Illumina platform were developed and tailored in‐house (SimcereDx). End repair was carried out using two different kits: the KAPA HyperPlus DNA Library Prep kit (Roche Diagnostics) and the VAHTSTM Universal DNA Library Prep Kit for Illumina® (Vazyme). Each step of the process was conducted in accordance with the protocols provided by the manufacturers. The concentration of the final DNA library was measured using the Qubit Fluorometer along with the Qubit dsDNA HS Assay kit (Thermo Fisher), while the library quality was assessed using the Agilent 4200 TapeStation system (Agilent). Prepared DNA libraries were sequenced on Illumina NovaSeq 6000 platform (Illumina). The fastp tool (V.2.20.0) was used for adapter pruning and to filter low‐quality sequencing reads.^27^ Cleaned reads were aligned to the human reference genome (hg19) using the burrows‐wheeler transform‐mem algorithm.^28^ Somatic mutations of 551 genes including point mutations, small insertions and deletions were identified and annotated using VarDict and InterVar, respectively.^29^, ^30^ We screened for germline variations using the internal database. Copy number variation involved amplification and deletion were analysed by CNVkit (dx1.1).^31^

## 3 RESULTS

### 3.1 A snRNA‐seq atlas in primary pulmonary sarcomas and pulmonary sarcomatoid carcinoma

**FIGURE 1:**
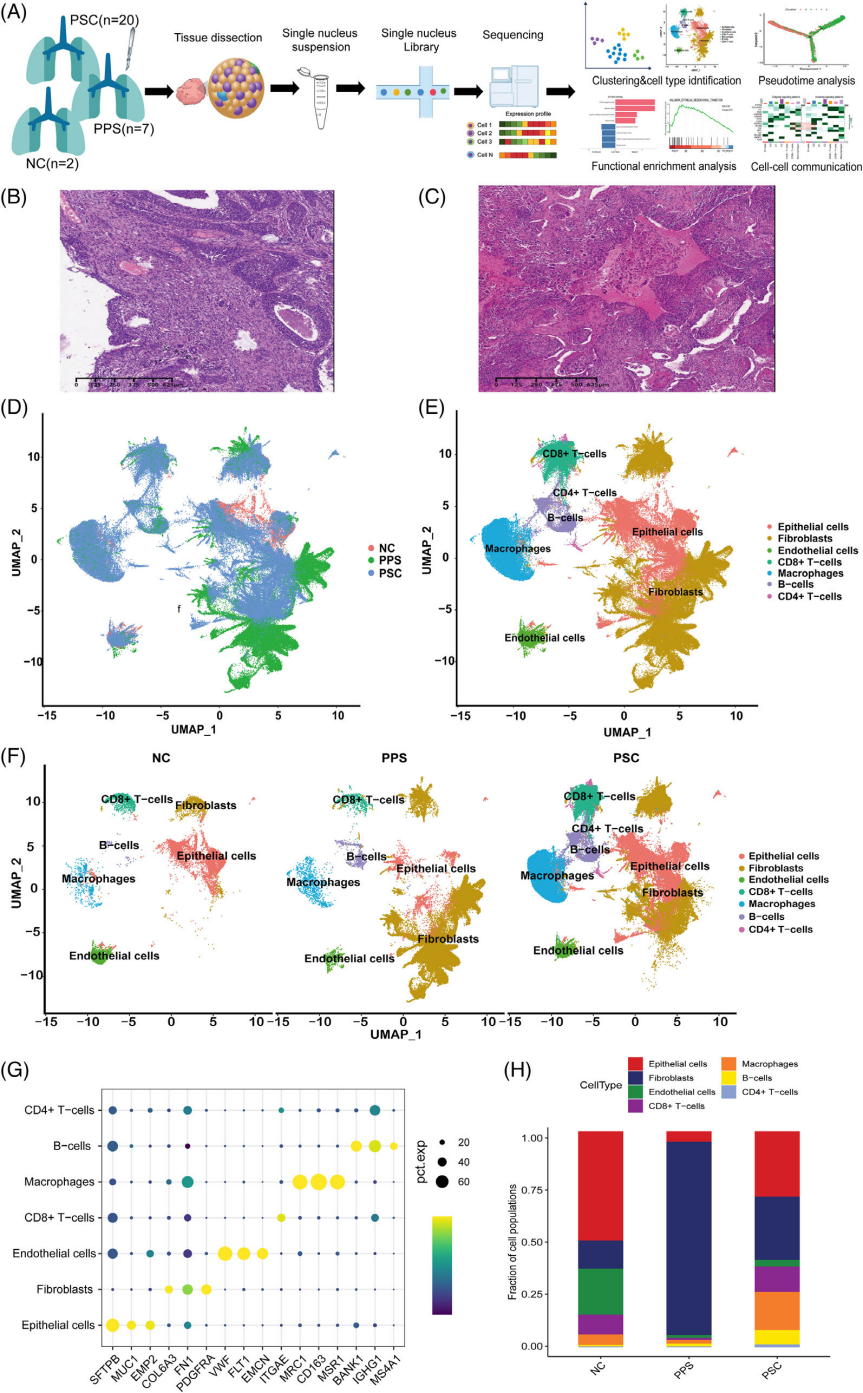
Cellular atlas of PPS and PSC. (A) Schematic diagram for the generation of snRNA‐seq data. 20 PSC samples, 7 PPS samples and 2 normal control (NC) samples were collected. (B) Typical HE characteristics of PSC. (C) Typical HE characteristics of PPS. (D) UMAP plots for the high‐quality cells showing sample pathological classification. (E) UMAP plots showing cell types for all samples. (F) UMAP plots showing cell types for NC (left), PPS (middle) and PSC (right). (G) The expression of corresponding marker gene for different cell types. (H) The proportion of each cell type in NC, PSC and PPS group.

**EXTENDED DATA FIGURE 2:**
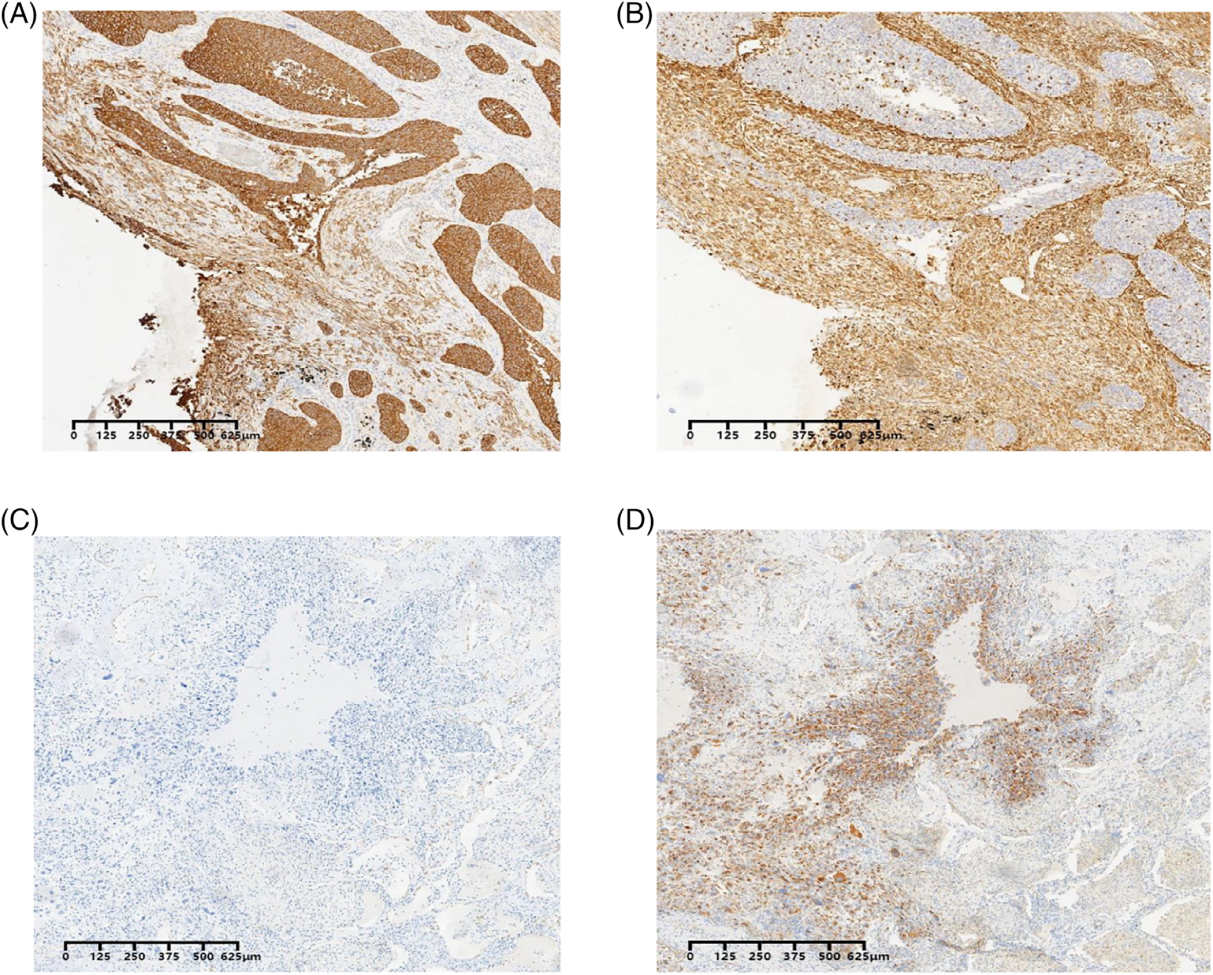
Typical IHC characteristics of PSC and PPS. (A) CK was positive in both epithelioid and spindle cells in PSC. (B) Vimentin was positive in spindle cells and negative in epithelioid cells in PSC. (C) CK was negative spindle cells in PPS. (D) Vimentin was positive in spindle cells in PPS.

**EXTENDED DATA FIGURE 3:**
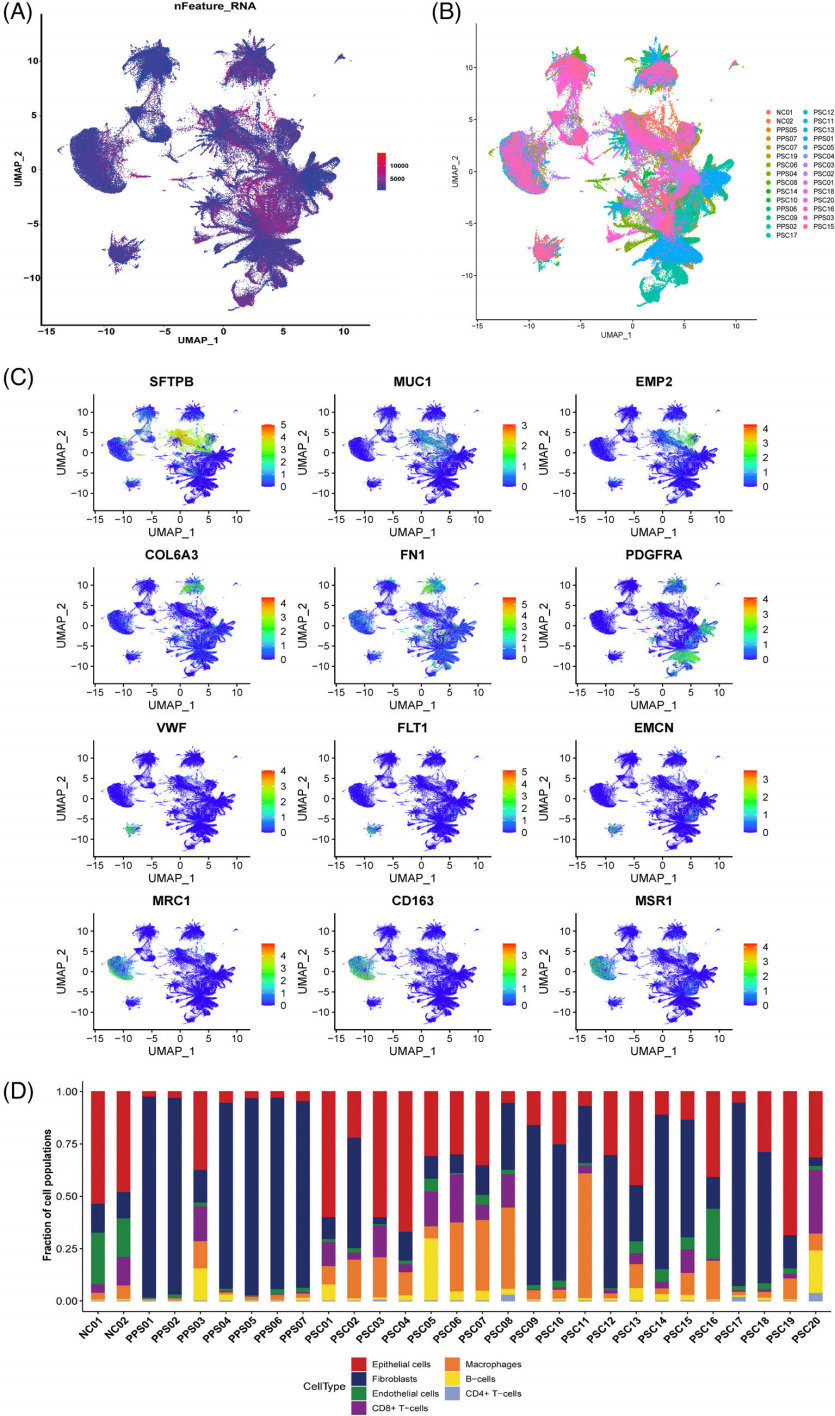
Cellular atlas in each sample of PPS and PSC. (A) UMAP of the cells profiled here (after removal of low‐quality cells, with each cell colorcoded for the number of genes (nGene) detected in each cell. (B) UMAP plots for the high‐quality cells showing each sample classification. (C) UMAP plots showing the expression levels of marker genes for each cell type. (D) The proportion of each cell type in each sample.

**FIGURE 2:**
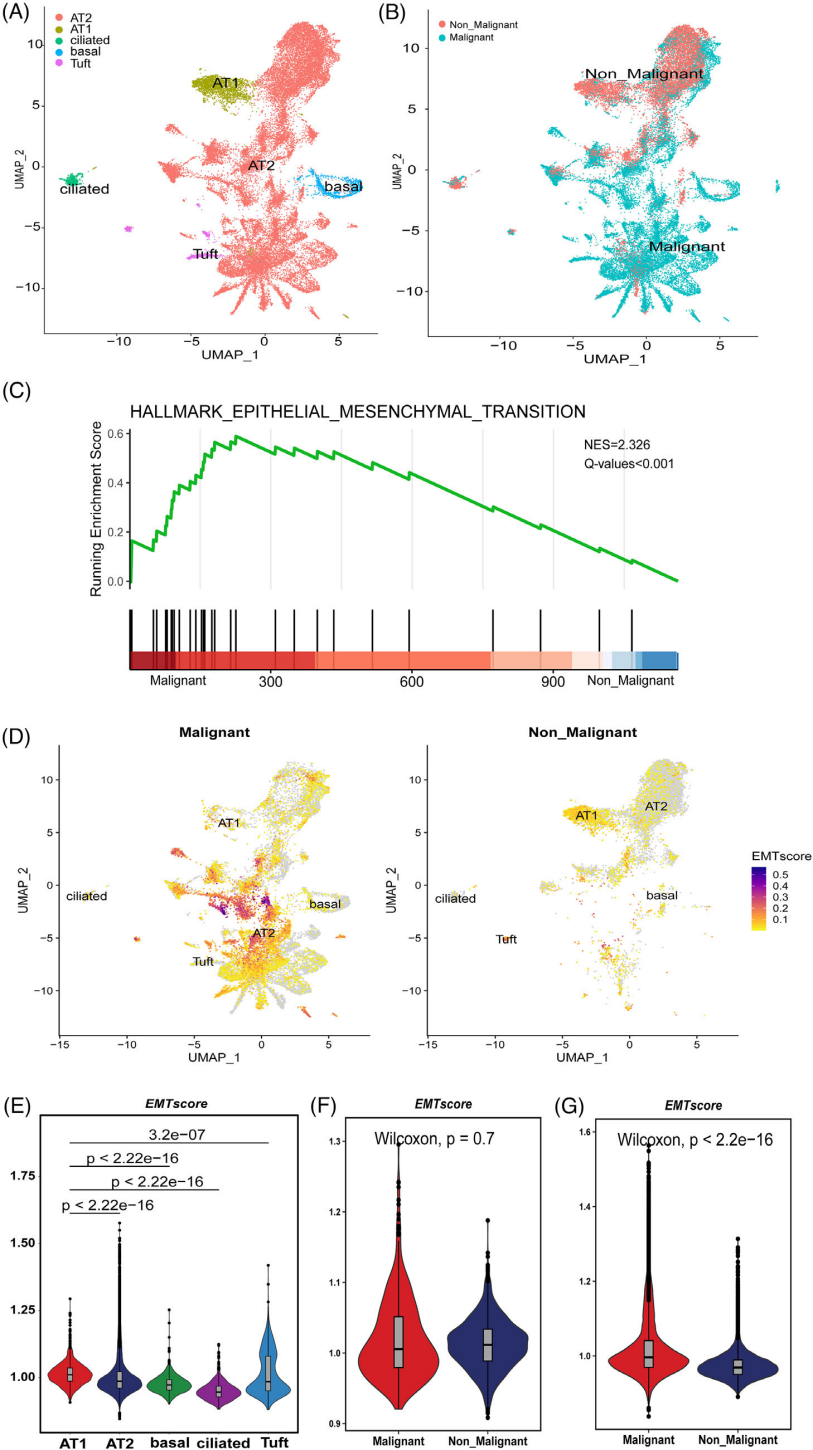
Classification of epithelial cells in PSC. (A) UMAP of PSC epithelial cells recluster. (B) Classification of malignant and non‐malignant epithelial cells in PSC. (C) GSEA analysis of malignant and non‐malignant epithelial cells; (D‐E) Comparison of the distribution and expression of EMT score in different types of epithelial cells; (F) Comparison of EMT scores between malignant and non‐malignant AT1 cells; (G) Comparison of EMT scores between malignant and non‐malignant AT2 cells.

To investigate the cell populations and the associated molecular characteristics between PSC and PPS, 20 PSC, seven PPS and two non‐malignant samples (normal control from adjacent tumour) were included in our survey (Figure 1A). The enrolled patients were all newly diagnosed and had not been diagnosed with any other tumours. The pathological type of all samples was reviewed by the pathologist (Figure 1B,C and Extended Data Figure 2). The clinical characteristics of these participants including age, gender, clinical stages, pathological features were recorded in Extended Data Table 2. After removal of low‐quality cells (see ‘Methods’ section), 181 764 cells were retained for biological analysis, which detected a median of 2059 genes (Extended Data Figure 3A,B). After normalisation of gene expression and principal component analysis (PCA), we used graph‐based clustering to partition the cells (Figure 1D–F). All cells were divided into seven clusters, including epithelial cells, fibroblasts, endothelial cells (ECs), CD8+T cells, macrophages, B cells, CD4+T cells (Figure 2E,F). These clusters could be assigned to known cell lineages through marker genes (Figure 1G and Extended Data Figure 3C). The proportion of each cell lineage varies greatly between PSC and PPS groups, epically in epithelium cells and fibroblasts. Compared with PPS group, PSC group had a higher proportion of epithelial cells (30.4% vs. 4.8%), but a lower proportion of fibroblasts (29.4% vs. 89.8%; Figure 1H and Extended Data Figure 3D). In addition, compared with PPS, PSC also has a higher proportion of CD8+ T cells. Our research group previously explored the immune microenvironment of PSC through IHC methods. Most patients with PSC had positive infiltration of CD8+ T cells in the tumour area, and had positive expression of PD‐L1 in tumour cells (Tumor cell proportion score ≥ 1).^32^ Notably, histologically, PPS is usually categorised as malignant fibrous histiocytoma, non‐epithelial tumour.^33^, ^34^ By analysing cell types, we discovered that there was still a very small proportion of epithelial cells (4.8%) in PPS.

**EXTENDED DATA TABLE 2:** Pathological characteristics of patients in two groups.

Variable		Pulmonary sarcomatoid carcinoma (PSC)		Primary pulmonary sarcomas (PPS)	
		*N*	%	*N*	%
Gender					
Male	21	16	76.2	5	23.8
Female	6	4	66.7	2	33.3
Age					
<70 years	20	14	70.0	6	30.0
≥70 years	7	6	85.7	1	14.3
Smoking status					
Non‐smokers	10	6	60.0	4	40.0
Smokers	17	14	82.4	3	17.6
Surgery					
Lobectomy	24	18	75.0	6	25.0
Pneumonectomy	3	2	66.7	1	33.3
pT stage					
T1	3	2	66.7	1	33.3
T2	11	8	72.7	3	27.3
T3	3	3	100	0	0
T4	10	7	70.0	3	30.0
pN stage					
N0	15	8	53.3	7	46.7
N1	2	2	100	0	0
N2	10	10	100	0	0
pTNM stage					
Stage I	3	2	66.7	1	33.3
Stage II	6	3	50.0	3	50.0
Stage III	18	15	83.3	3	16.7
Adjuvant therapy					
With	16	12	75.0	4	25.0
Without	11	8	72.7	3	27.3

This result indicates that PSC and PPS harbour distinct Cell composition on a single‐cell level. Therefore, further examination of epithelial cells and fibroblasts may reveal the origin of PPS and PSC.

### 3.2 Classification of malignant and non‐malignant epithelial cells in PSC and PPS

**EXTENDED DATA FIGURE 4:**
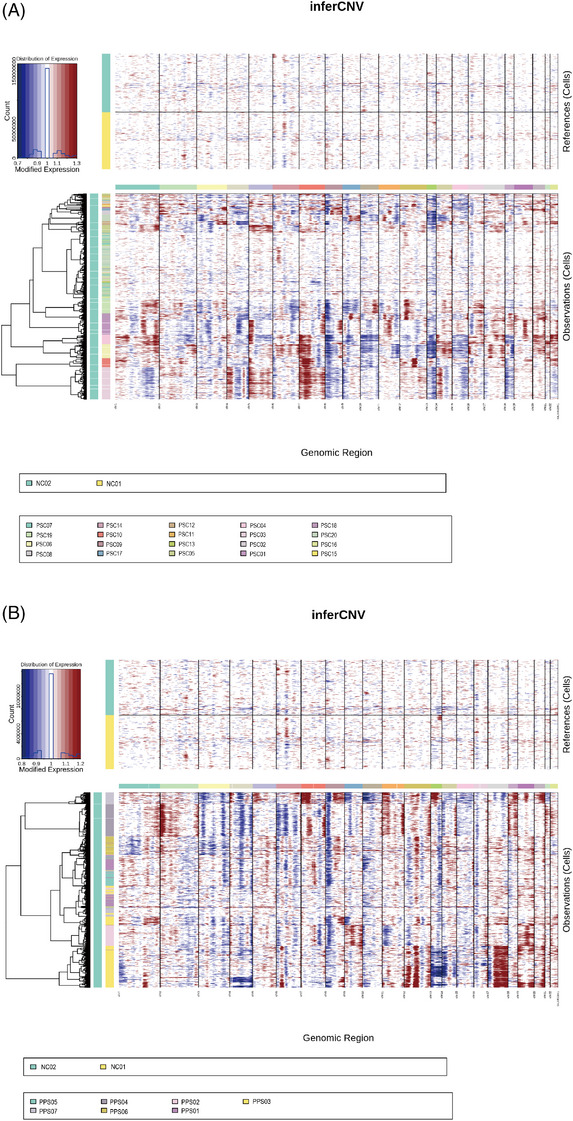
Identification of malignant and non‐malignant cells. Inferred large‐scale copy number variations (CNVs) was used to distinguish between malignant and non‐malignant cells in PSC (A) and PPS (B) group. Epithelial cells and fibroblasts in normal control are included in the x axis and chromosomal regions on the y axis. Amplifications (red) or deletions (blue) were inferred by averaging expression over 100‐gene stretches on the respective chromosomes.

**EXTENDED DATA FIGURE 5:**
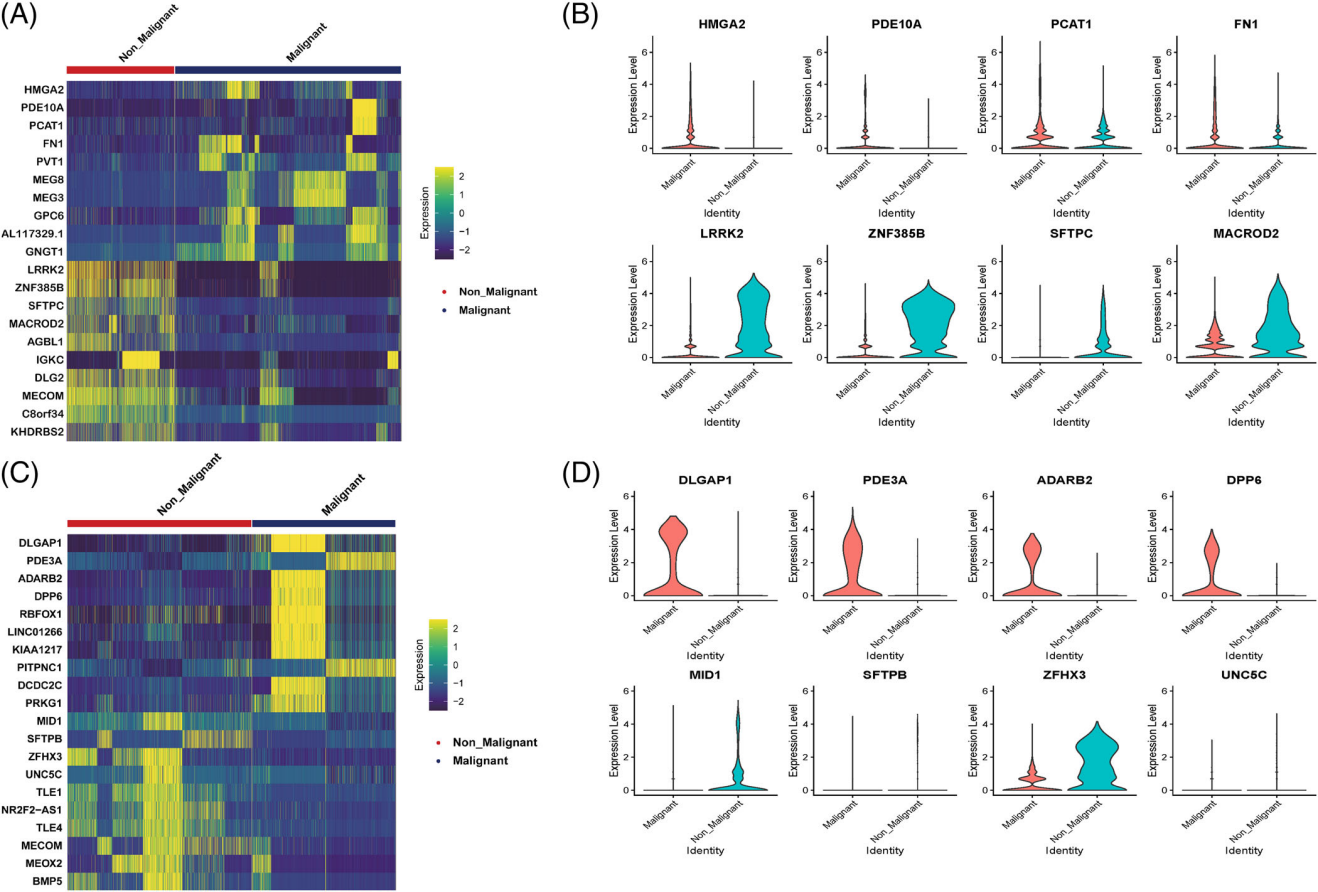
DEGs expression of malignant and non‐malignant epithelial cells. (A) Heatmap showing DEGs between malignant and non‐malignant epithelial cells in PSC group. (B) UMAP showing the expression levels of DEGs for each cell type in PSC group. (C) Heatmap showing DEGs between malignant and non‐malignant epithelial cells in PPS group. (D) UMAP showing the expression levels of DEGs for each cell type in PPS group.

To identify distinct transcriptional profiles of epithelial cell populations, we employed dimensionality reduction and unsupervised cell clustering techniques using the Seurat package. By leveraging key marker genes expression, we successfully reclustered epithelial cells of PSC into five major cell types, comprising AT1 cells (AGER; TIMP3), AT2 cells (SFTPB; SFTPA1), ciliated cells (TPPP3; RSPH1), and basal cells (KRT15; KRT17) (Figure 2A). In the epithelial cells of PSC, AT2 cells accounted for the vast majority. Additionally, in order to distinguish between malignant and non‐malignant epithelial cells in PSC and PPS, we employed CNV analysis of RNA expression due to the established correlation between cancer development and large‐scale chromosomal alterations.^35^ In comparison to ECs in the control group, cancer cells exhibited more pronounced alterations in relative expression intensities across the entire genome (Extended Data Figure 4). Among the malignant epithelial cells, the vast majority are AT2 cells (Figure 2B). By comparing malignant and non‐malignant epithelial cells in PSC, malignant cells showed high expression of marker genes associated with epithelial–mesenchymal transition (EMT) and tumour metabolism regulation related genes, such as HMGA2, FN1, PDE10A, and PCAT1 (Extended Data Figure 5A,B). Referring to the EMT‐related gene set in Hallmark gene set (including 200 genes),^36^ we conducted pathway enrichment through GSEA. The results indicated that EMT pathway genes were highly expressed in malignant epithelial cells (Figure 2C). Using the genes in this gene set as marker genes and referring to the methods in the literature, the EMT score values of different types of cells in PSC were evaluated. The results indicated that the EMT scores of different types of epithelial cells varied significantly (Figure 2D,E). It is worth noting that for AT1 cells, there was no statistically significant difference in EMT scores between malignant and non‐malignant cells (Figure 2F). For AT2 cells, malignant AT2 cells significantly have a higher EMT score. This result demonstrates that malignant AT2 cells in PSC have a higher ability for EMT (Figure 2G).

**FIGURE 3:**
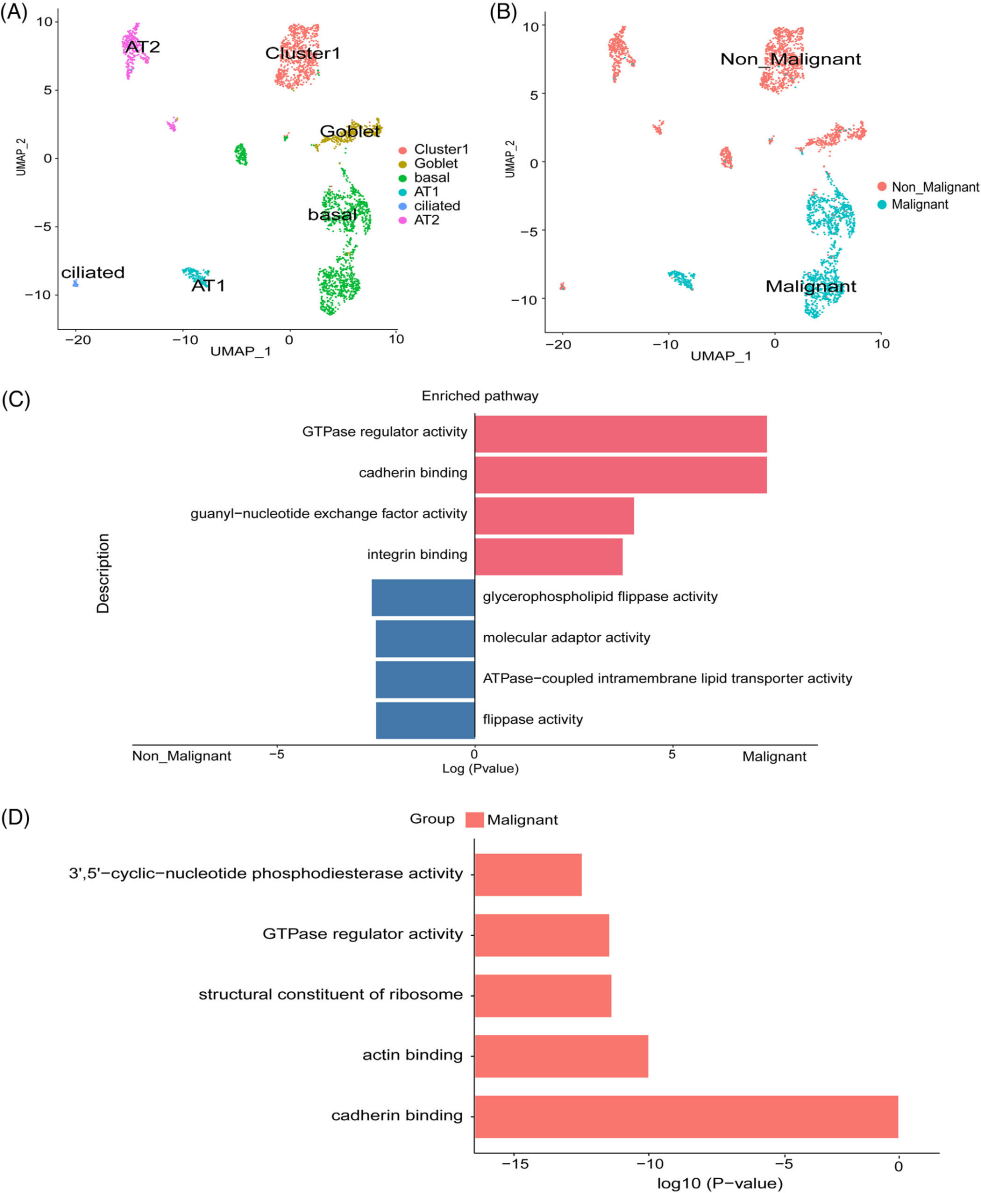
Classification of epithelial cells in PPS and enriched pathway in PSC and PPS. (A) UMAP of PPS epithelial cells recluster. (B) The classification of malignant and non‐malignant epithelial cells in PPS. (C‐D) Bar plot showing enriched pathway in malignant cells and non‐malignant epithelial cells in PSC (C) and PPS (D).

While in the PPS group, six cell types were identified, including five known cell type AT1 cells, AT2 cells, ciliated cells, basal cells and goblet cells (NPDC1) and one unknown cell type cluster 1 (Figure 3A). AT1 cells and basal cells in PPS groups were likely to malignant epithelial cells (Figure 3B). Previous single‐cell studies have demonstrated that AT2 cells are the source of lung adenocarcinoma,^37^, ^38^ AT1 cells are an important driver of lung squamous cell carcinoma.^39^ In our study, the AT2 cells of PPS were identified as non‐malignant cells, while AT1 cells were recognised as malignant cells. The malignant epithelial cells in the PPS group exhibited elevated expression of genes involved in metabolic regulation, such as DLGAP1 and PDE3A, as well as heightened expression of neuronal regulatory genes, including ADARB2 and DPP6 (Extended Data Figure 5C,D). To decipher the molecular characteristics difference of malignant and non‐malignant epithelium, we performed GSEA. Compared with non‐malignant epithelium, malignant epithelium in PSC was enriched for signalling pathways in integrin binding, guanyl–nucleotide exchange factor activity, actin binding, cadherin binding, GTPase regulator activity (Figure 3C). Malignant epithelium in PPS was also enriched for actin binding, cadherin binding, GTPase regulator activity. Moreover, malignant epithelium in PPS was enriched for 3′,5′‐cyclic‐nucleotide phosphodiesterase activity and structural constituent of ribosome (Figure 3D).

### 3.3 The epithelial cell repertoire of PSC is markedly changed and contains aberrant AT2 cells

**FIGURE 4:**
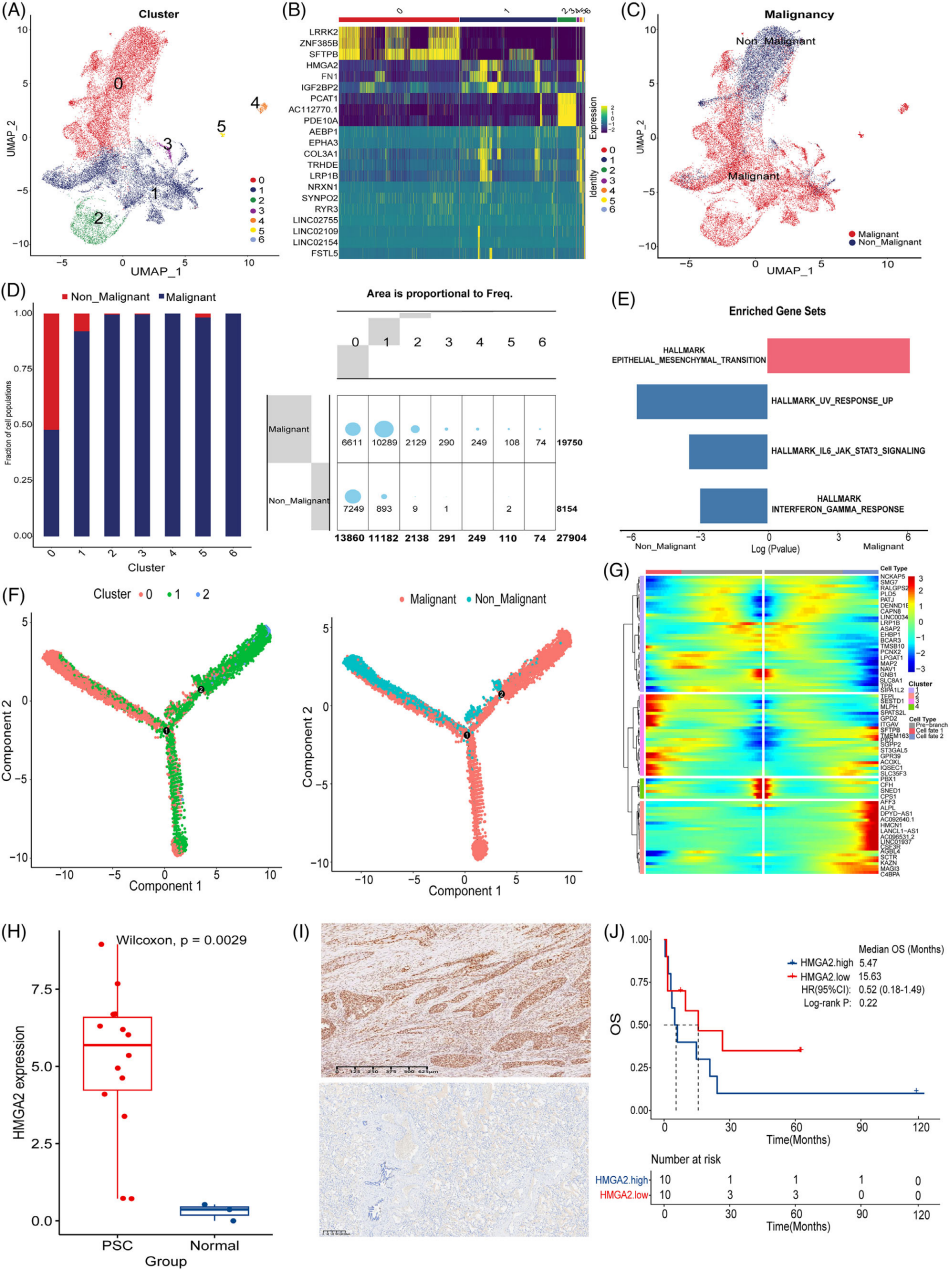
The HMGA2+ subtype cells contributed to the malignant progression of PSC. (A) UMAP of AT2 cells recluster. (B) Heatmap showing DEGs among AT2 each cluster. (C) The classification of malignant and non‐malignant AT2 cells. (D) The proportion of each cell cluster of AT2 cells. (E) Bar plot showing enriched pathway in malignant cells and non‐malignant AT2 cells. (F) Pseudotime analysis by Monocle2 shows the potential evolutionary trajectory of AT2. (G) According to the differential expression gene heatmap of the 3 different branches (fate 1, fate 2 and pre‐branch), the genes were divided into 4 main gene modules (Cluster 1, 2, 3, 4), which extended from the center to the left and right into 2 cell fates, as shown by the red arrow. Red indicated upregulation; blue indicated downregulation. (H) Comparison of HMGA2 expression in PSC samples and normal controls in GEO database. (I) Immunohistochemistry (IHC) result of HMGA2 positive stain in representative PSC (up) sample and control (down). (J) The HMGA2 expression level and survival analysis.

**EXTENDED DATA FIGURE 6:**
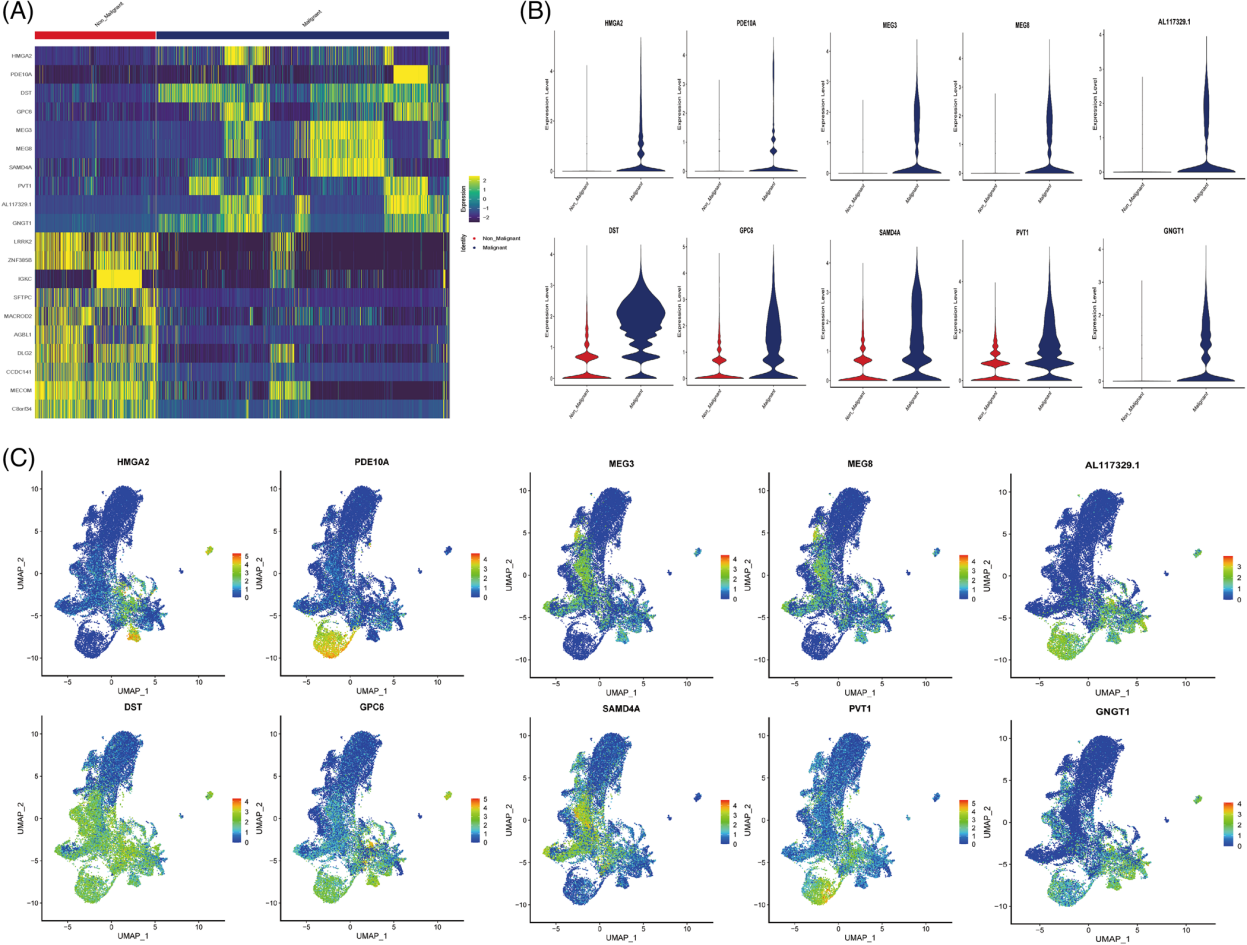
The DEGs expression of malignant and non‐malignant AT2 cells in PSC. (A) Heatmap showing DEGs between malignant and non‐malignant AT2 cells. (B) Violin plots showing the expression of representative genes with differential expression between malignant and non‐malignant AT2. (C) UMAP showing the expression levels of DEGs between malignant and non‐malignant AT2 cells.

The vast majority of epithelial cells in PSC were comprised AT2 cells. Unsupervised cell clustering revealed the AT2 cells could be further divided into seven clusters (Figure 4A). Because of the cell numbers of cluster (cluster 3–cluster 6) being insufficient, we proceeded to investigate cluster 0–cluster 2 (C0–C2). The C0 cluster overexpressed LRRK2, a gene associated with lung homeostasis, which was highly expressed in normal lungs, as well as SFTPB, marker of the common AT2 phenotype.^40^ The C1 cluster highly expressed HMGA2, which was upstream mediator of cancer hallmarks and targets many critical signalling pathways.^41^ The C1 cluster also highly expressed EMT marker FN1. The C2 expressed enzyme activity related genes, such as PCAT1 and PDE10A (Figure 4B).^42^, ^43^ The differential expression genes from C0 to C2 cluster suggested a phenotypic transition towards malignancy. This result was consistent with the proportion of malignant cells identified by different AT2 cells (Figure 4C,D). Moreover, the C1 and C2 highly expressed HMGA2 and PDE10A, which was enriched in malignant AT2 cells (Extended Data Figure 6). The GSEA analysis revealed that malignant AT2 cells exhibited upregulated of the EMT pathway and downregulated of INF‐γ, IL‐6_JAK_STAT3 and UV response pathway (Figure 4E). Previous research has indicated that AT2 cells, through a process of dedifferentiation, enhance stemness and facilitate EMT, thus contributing to the progression of pulmonary adenocarcinoma.^37^ Our results also elucidated the similar significant role of AT2 cells in PSC. Pseudotime analysis by Monocle2 shows the potential evolutionary trajectory of AT2 cells (Figure 4F). Dynamically expressed genes were screened depended on the regulation of the branch during the transition from pre‐branch to cell‐fate 1 and cell‐fate 2 branches (Figure 4G) and the genes were divided into four clusters (cluster 1–4). Most of the genes in cluster 1 and cluster 2 are highly expressed in the pre‐branch, and their functions are involved in transcription and development, such as PTPN14, PBX1 and BZW1.^44^, ^45^, ^46^ The genes in cluster 3 exhibited high expression levels in cell fate 1, which were associated with tumourigenesis, EMT and other processes, such as ROR1.^47^ Given the important role of transcriptional regulation and EMT in malignant transformation of AT2 cells, and the important position of HMGA2 in these biological processes, we speculated that HMGA2 is an important marker of malignant AT2 cells. Therefore, the HMGA2 expression levels of PSC samples in gene expression omnibus (GEO) database (GSE110205) and normal controls were compared. The results showed that HMGA2 was highly expressed in PSC samples (*p* = .0029, Figure 4H). We also verified the results at the protein level by IHC (Figure 4I). In addition, despite not reaching statistically significant differences, PSC patients in our cohort with high HMGA2 expression had a worse prognosis in our cohort (mOS: 5.47 vs. 15.63 months, *p* = .22, Figure 4J).

**FIGURE 5:**
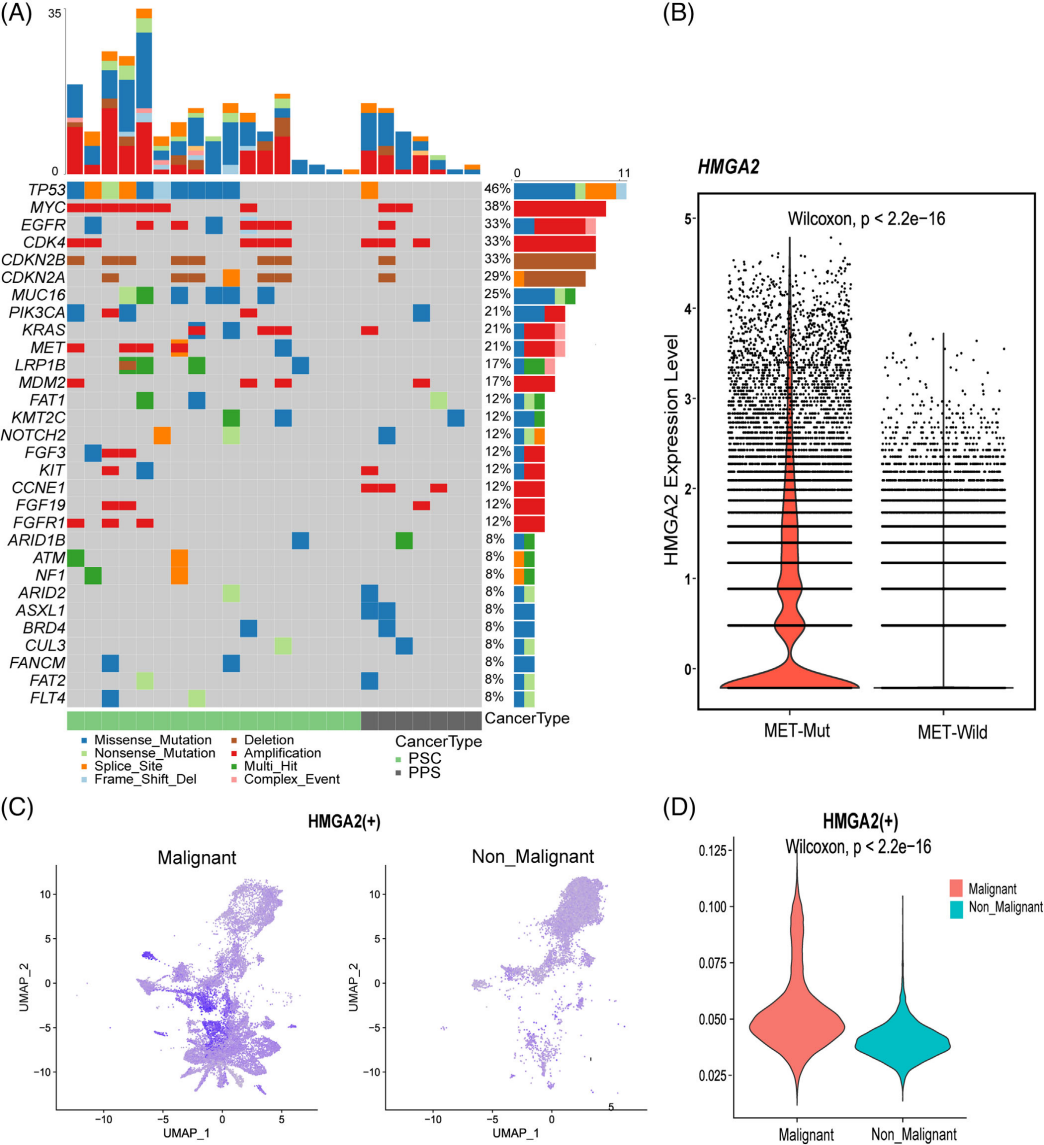
Relationship between HMGA2 and driver genes as well as transcription factor analysis. (A) mutation landscape of all samples (17 PSC and 7 PPS); (B) The expression of HMGA2 under different MET mutation status; (C) tSNE plots of the expression levels of transcription factor HMGA2; (D) Comparison of Transcription Factor Scores of HMGA2 in malignant and non‐malignant AT2 cells.

MET gene has been confirmed as a driver gene in PSC.^48^ Previous studies on NSCLC have suggested that patients with MET mutations may be more likely to have HMGA2 amplification.^49^, ^50^ We performed DNA‐based next‐generation sequencing sequencing on PSC and PPS. Among the 17 cases of PSC, five cases had MET mutations (Figure 5A). Moreover, patients with MET mutations have higher levels of HMGA2 expression (Figure 5B). Meanwhile, HMGA2, as a transcription factor, is highly expressed in malignant AT2 cells (Figure 5C,D).

### 3.4 Classification of malignant and non‐malignant fibroblasts and endothelial cells in PSC and PPS

**FIGURE 6:**
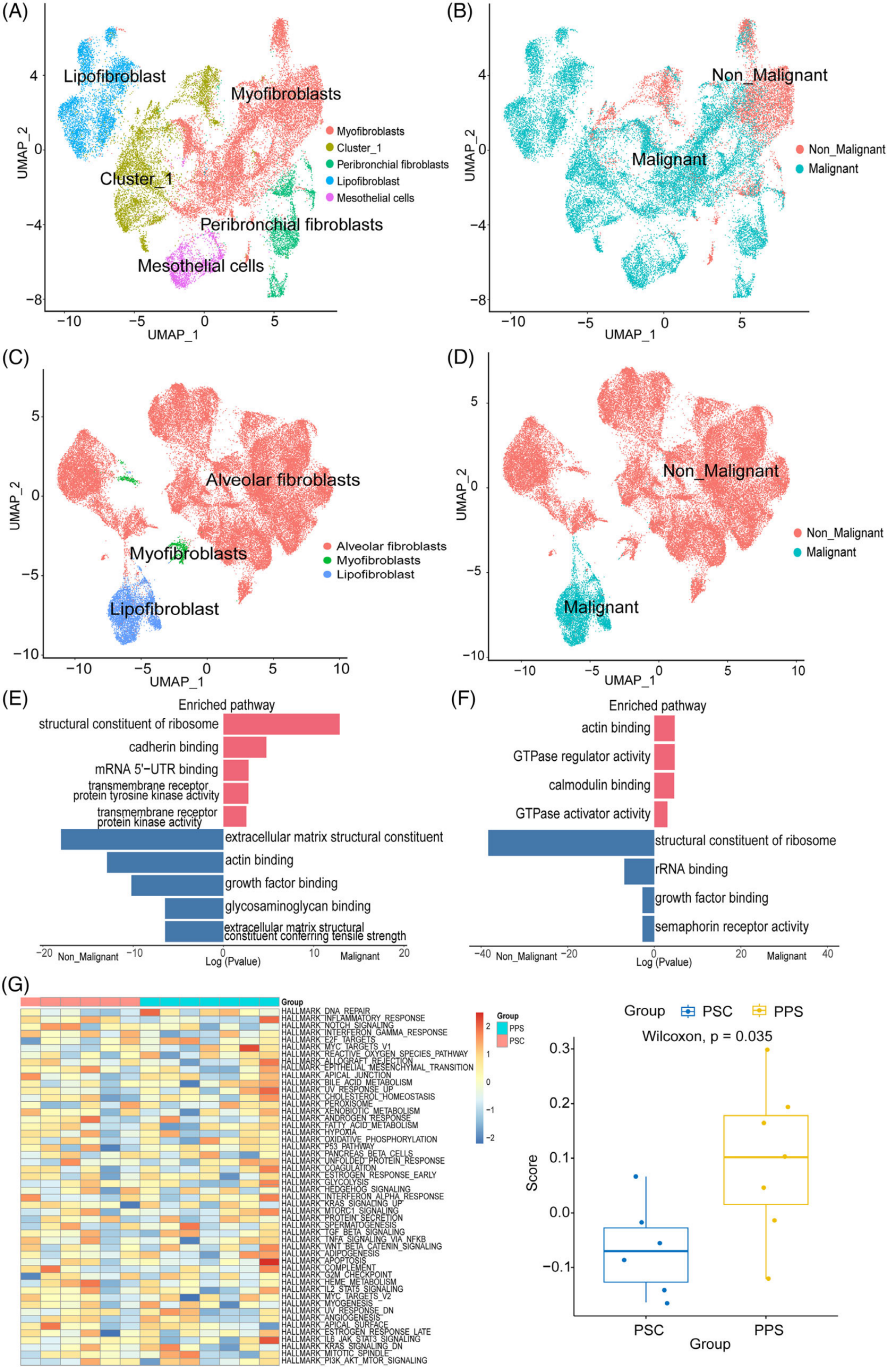
Classification of fibroblasts in PSC and PPS. (A) UMAP of PSC fibroblasts recluster. (B) Classification of malignant and non‐malignant fibroblasts in PSC. (C) UMAP of PPS fibroblasts recluster. (D) The classification of malignant and non‐malignant fibroblasts in PPS. (E‐F) Bar plot showing enriched pathway in malignant cells and non‐malignant fibroblasts in PSC (E) and PPS (F) group. (G) Differences in pathway activities scored of lipofibroblasts by GSVA between PSC and PPS group (left). Comparison of GSVA score in DNA repair pathway between PSC and PPS group (right).

**EXTENDED DATA FIGURE 7:**
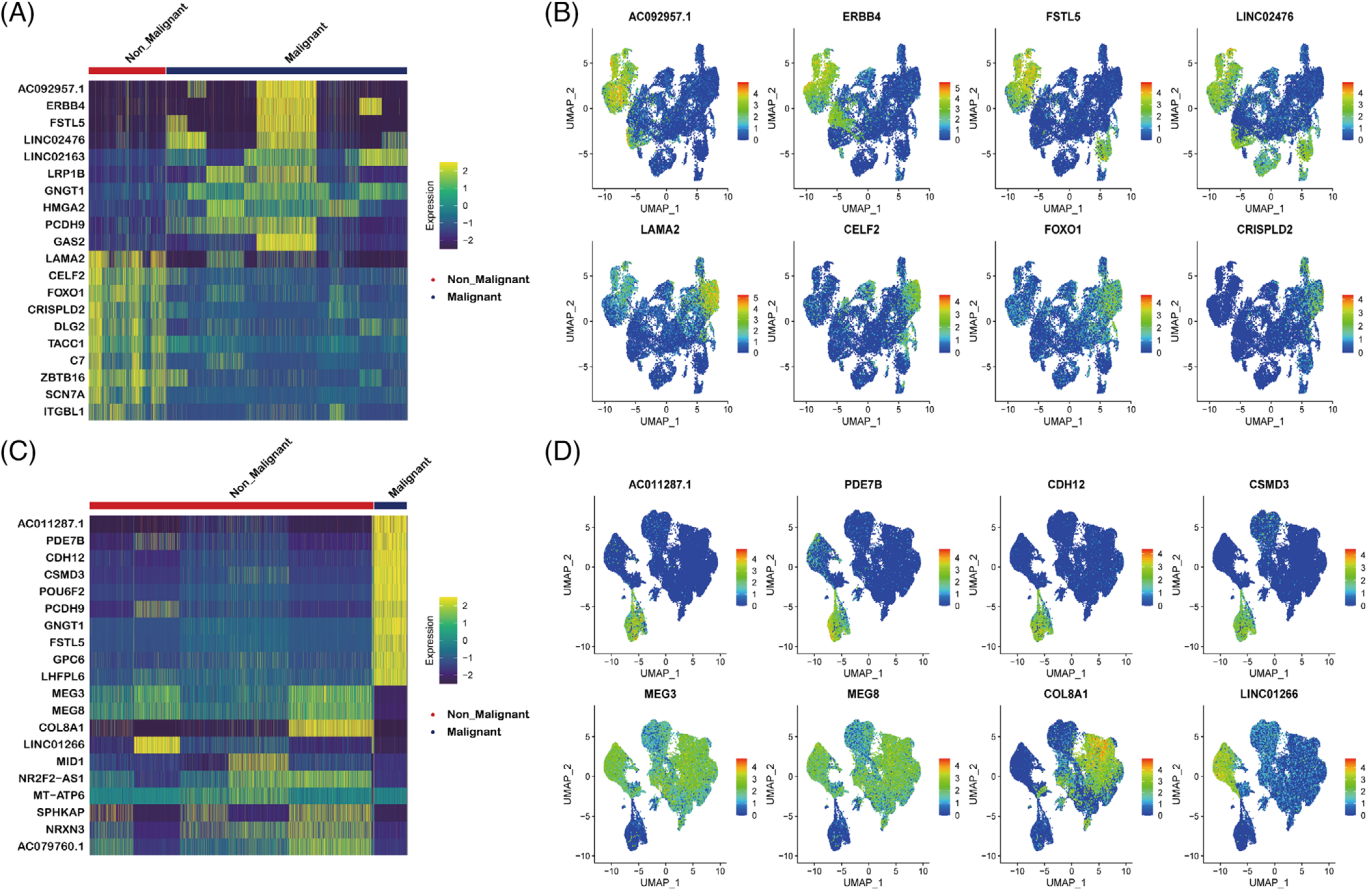
The DEGs expression of malignant and non‐malignant fibroblasts in PSC and PPS. (A) Heatmap showing DEGs between malignant and non‐malignant fibroblasts in PSC group. (B) UMAP showing the expression levels of DEGs for each cell type in PSC group. (C) Heatmap showing DEGs between malignant and non‐malignant fibroblasts in PPS group. (D) UMAP showing the expression levels of DEGs for each cell type in PPS group.

By utilising key marker gene expression, we reclassified fibroblasts into five major cell types, comprising myofibroblasts, peribronchial fibroblasts, lipofibroblasts, mesothelial cells and cluster 1 (Figure 6A). In PSC group, most of the fibroblasts were malignant (Figure 6B). In comparison to fibroblasts in the normal control, malignant cells also exhibited more pronounced alterations in relative expression intensities across the entire genome with inferCNV (Extended Data Figure 4). By comparing malignant and non‐malignant fibroblasts in PSC, malignant cells showed high expression of marker genes associated with EMT and tumour signal transduction, such as FSTL5 and ERBB4 (Extended Data Figure 7A,B).^51^, ^52^ While in the PPS group, three cell subtypes were identified, including alveolar fibroblasts, myofibroblasts and lipofibroblasts (Figure 6C). Notably, in the PPS group, only lipofibroblasts showed the characteristics of malignant cells (Figure 6D). The malignant fibroblasts in PPS exhibited upregulation of the EMT‐related gene CDH12, as well as upregulation of apoptosis‐related genes such as MEG3 and MEG8 (Extended Data Figure 7C,D). The malignant cells in PSC exhibited a higher abundance of signalling pathways associated with transmembrane receptor protein kinase activity, among others, when compared to non‐malignant fibroblasts (Figure 6E). Malignant fibroblasts in PPS were enriched for GTPase‐related pathway (Figure 6F). The lipofibroblasts were malignant cells in both PSC and PPS groups. Therefore, A direct comparison of PSC versus PPS lipofibroblasts revealed the expression signature of both groups were similar, with the exception of an enrichment in the DNA repair pathway observed in lip fibroblasts of PPS (Figure 6G).

**FIGURE 7:**
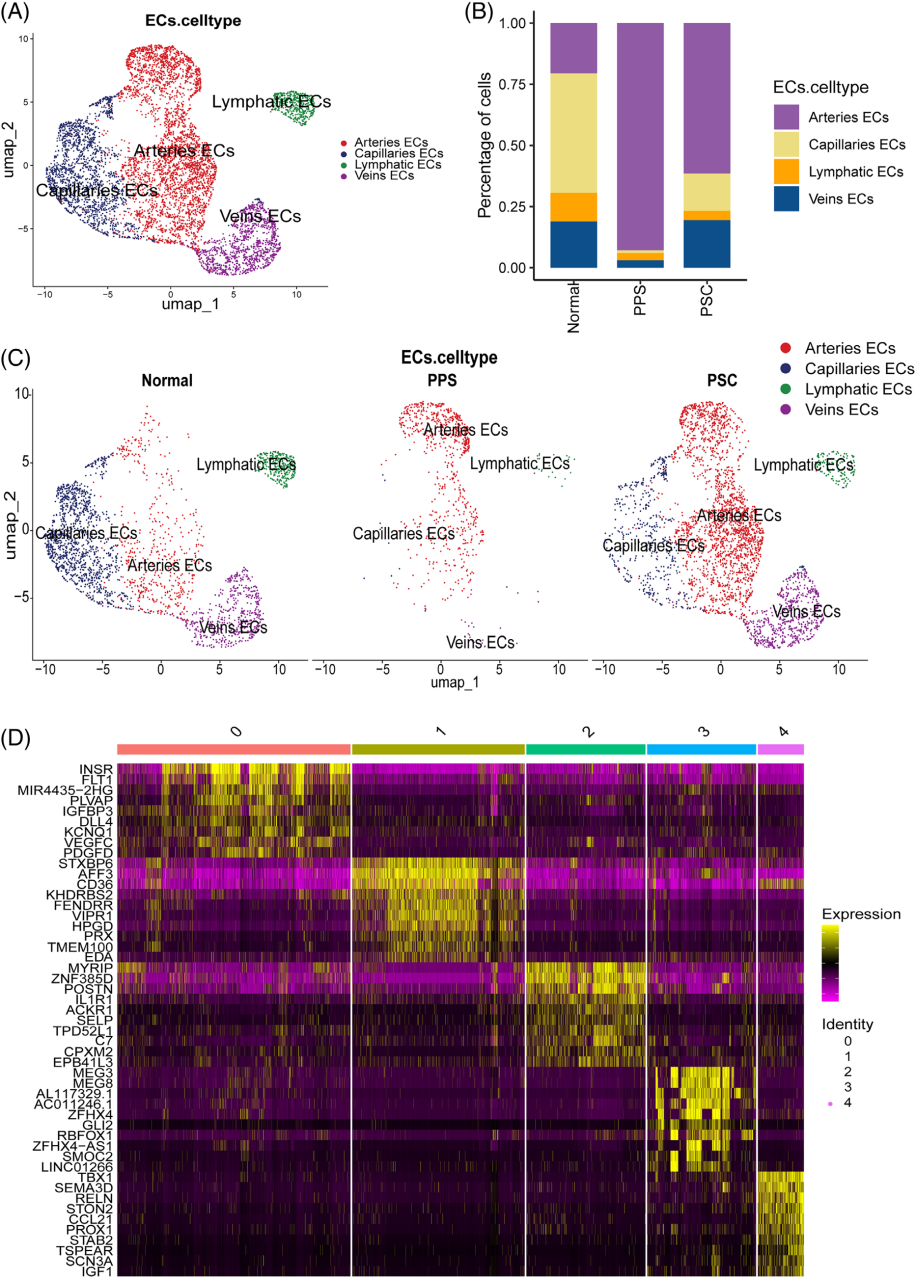
Classification of endothelial cells (ECs) in PSC and PPS. (A) UMAP of all samples ECs recluster. (B) The proportion of different subtypes of ECs; (C) The distribution of different subtypes of ECs in normal, PSC and PPS. (D) Differential gene expression in different types of ECs

Tumour angiogenesis represents a crucial characteristic of cancer, serving as a mechanism through which tumours secure the necessary nutrients and oxygen to support their growth.^53^ ECs serve as crucial elements of the vascular system and play a direct role in the formation of new blood vessels during tumour angiogenesis.^54^ To dissect the tumour vascular microenvironment, we recluster the ECs and annotated the cell types on the basis of representative gene signatures (Figure 7).^55^ The results indicated that PSC had the largest number of arterial ECs.

### 3.5 Inference of cell–cell communications in TME

**EXTENDED DATA FIGURE 8:**
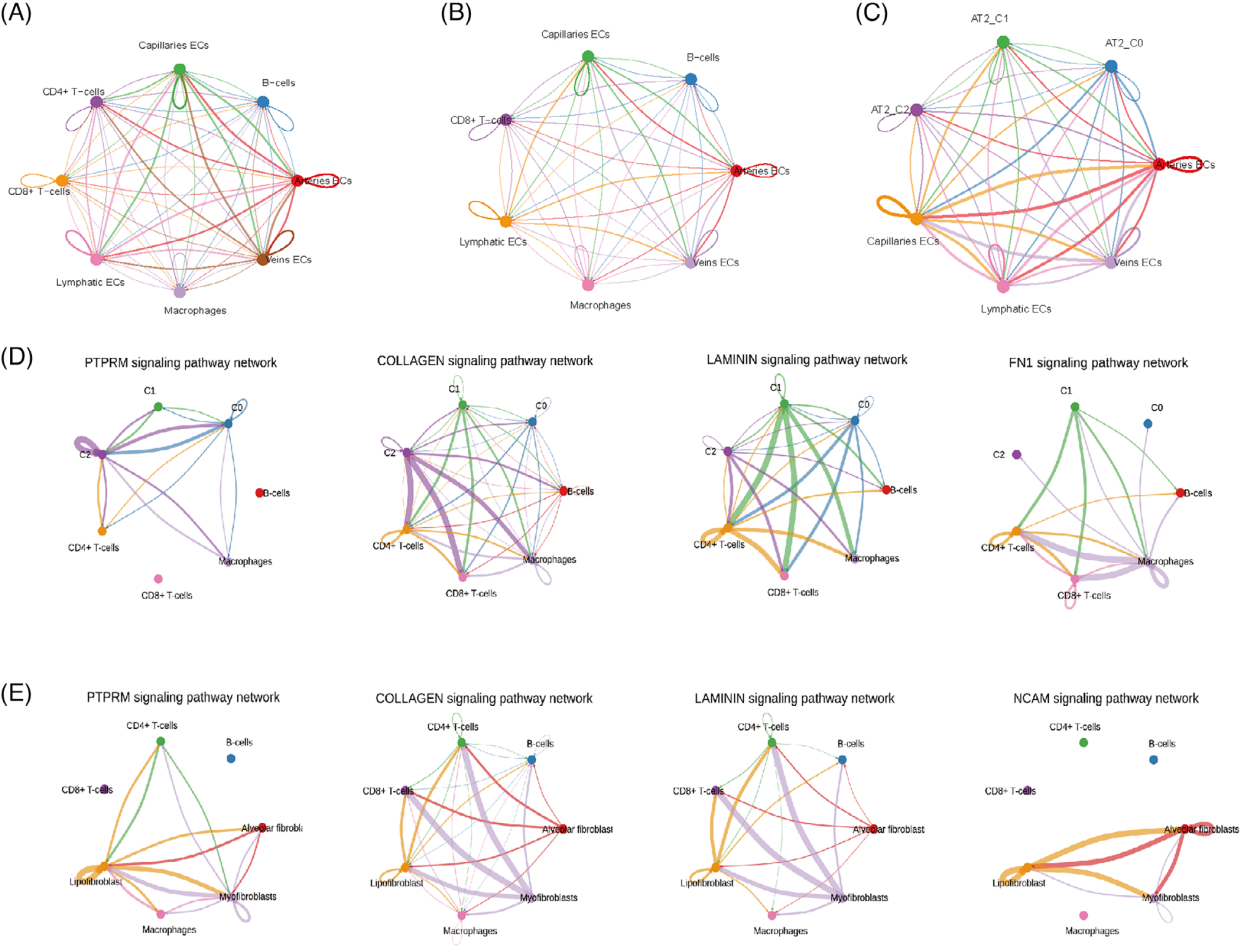
The cell–cell communication and interaction network among different cell types. (A) The results of cell‐cell communication among different types of endothelial cells in PSC; (B) The results of cell‐cell communication among different types of endothelial cells in PPS; (C) The results of cell‐cell communication between different types of AT2 cells and endothelial cells in PSC; (D) In PSC, the interaction networks among various cell types in different signaling pathways; (E) In PPS, the interaction networks among various cell types in different signaling pathways.

**FIGURE 8:**
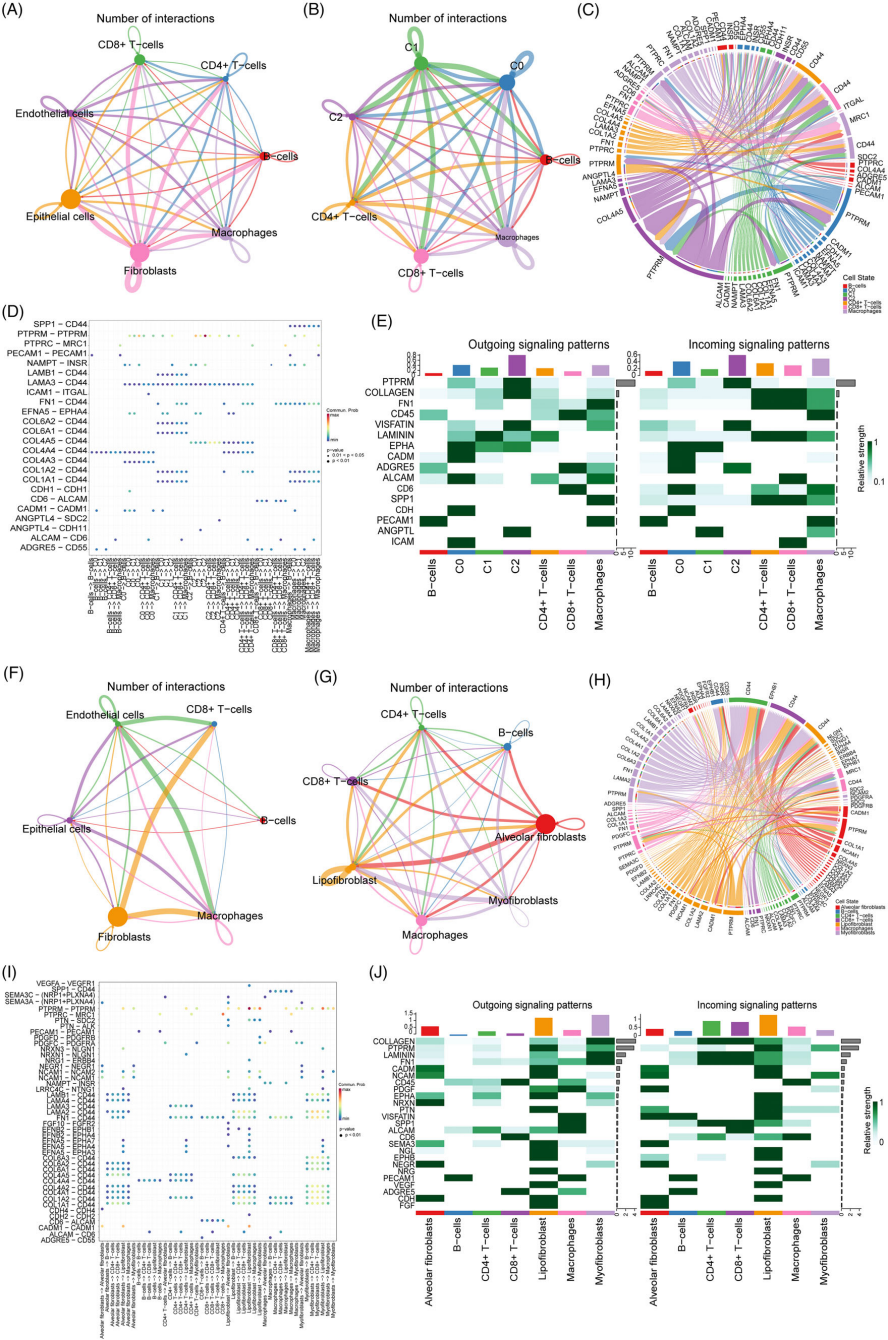
Cell–cell communication analysis in PSC and PPS. (A) Cell–cell communications between the identified cell types in PSC. (B) Cell–cell communications between different AT2 cells subtypes (cluster 0‐2, C0‐2) and immune cells in PSC. (C) Highly communicated ligand‐receptor interactions between AT2 cells (C0‐2) and immune cells in PSC. (D) Bubble heatmap showing the mean attraction strength for selected ligand‐receptor pairs. Dot size indicates P‐value generated by permutation test, colored by attraction strength levels (E) The incoming and outgoing signaling pathways of AT2 recluster and immune cells in PSC. (F) Cell–cell communications between the identified cell types in PPC. (G) Cell–cell communications between each type of fibroblasts and immune cells in PPS. (H) Highly communicated ligand‐receptor interactions between each type of fibroblasts and immune cells in PPS. (I) Bubble heatmap showing the mean attraction strength for selected ligand‐receptor pairs in PPS. (J) The incoming and outgoing signaling pathways of each type of fibroblasts and immune cells in PPS.

Given the variations of different cell type compositions within TME, it may be due to the alteration of the complex intercellular communications. Herein, we employ the CellChat which is specifically designed for quantitative inference of intercellular, communication using snRNA‐seq data, to discern cell–cell interactions and explore their potential implications in promoting tumour progression. Cell communication between different types of ECs and immune cells in PSC and PPS is shown in Extended Data Figure 8A,B. The numbers and strength of interactions among different cell populations in PSC are presented in Figure 8A. Given the significant role of epithelial cells, especially epithelial AT2 cells, in PSC, we further conducted CellChat between different AT2 subclusters (AT2‐C0, AT2‐C1, AT2‐C2) and immune cells. Once the interaction strength is considered (represented by the interaction counts), AT2‐C1 cells demonstrate a pivot role in the TME, which strongly interacts with other AT2 cells and immune cell types (Figure 8B). The AT2 subcluster also interacts with various types of ECs. However, more frequently, it is the communication among different types of ECs (Extended Data Figure 8C). We further explore the ligand–receptor pairs involved in the cellular crosstalk between AT2 cells and other immune cells (Figure 8C,D). Noteworthy, strong communication probabilities mostly occur between ligands including protein tyrosine phosphatases (PTPRM, PTPRC), collagens (type I and IV) and fibronectin (FN1), with receptors PTPRM, MRC1 and CD44. These ligands and receptors dominate the active signalling between AT2 and other cell types. The receptor CD44 is notably shared by all cell types, making it the most prevalent one. While its association with the TME is well‐known, this study provides the first documentation of its role as a common receptor in PSC. To elucidate the signalling pathways contributing to intricate intercellular communication, we quantitatively assess both the outgoing and incoming interaction strengths of each individual pathway. Notably, PTPRM signalling is shown as the most predominant signalling in AT2‐C2, as reflected by the largest outgoing and incoming interaction strength compared to other signalling pathways. In addition to PTPRM signalling, we also observe increased collagen, extracellular matrix (ECM) signalling related to fibronectin 1 (FN1) and laminin (LAMININ) signal (Figure 8E and Extended Data Figure 8D,E). In PPS group, most interactions are observed among fibroblasts, CD8+T cells and macrophages (Figure 8F). Further, the cell communication between different types of fibroblasts and immune cells was analysed. Lipofibroblasts play a crucial role in the TME, interacting strongly with various cell types, particularly alveolar fibroblasts (Figure 8G). Ligand–receptor analysis revealed strong communication probabilities mostly occur between ligands including PTPRM, PTPRC, collagens type I, collagens type IV, LAMA2, LAMA3, LAMA4, LAMB1 and CADM1, with receptors PTPRM, MRC1, CADM1 and CD44 (Figure 8H,I). The analysis of outgoing and incoming interactions showed collagen signalling was the mainly signalling in T cells and myofibroblasts (Figure 8J).

## 4 DISCUSSION

PPS and PSC are rare, malignant, sarcomatous subtypes of lung cancer. The diagnosis, treatment and prognosis of PSC and PPS all pose challenges. In this work, relying on snRNA‐seq, we investigated the single‐cell landscape of PSC and PPS. In clinical practice, the clinical manifestations and imaging of PSC and PPS are similar. At present, extensive tissue sampling is the key to distinguishing PCS from PPS. While, at the single‐cell level, we identified significant disparities in cell composition between PSCs and PPSs. On the whole, PSC group had a higher proportion of epithelial cells (30.4% vs. 4.8%), but a lower proportion of fibroblasts (29.4% vs. 89.8%) than PPS. According to WHO classification, PSC, as a poorly differentiated NSCLC, should contain at least a 10% component of spindle and/or giant cells, or a carcinoma that consists entirely of spindle and/or neoplastic giant cells.^56^ Therefore, there are considerable variations in the proportion of cells among different individuals of PSC. In addition, PPS is usually categorised as mesenchymal malignancy, non‐epithelial tumour.^7^ By analysing cell types, we discovered that there was still a very small proportion of epithelial cells (4.8%) in PPS. Through further exploration of the epithelial cells of PPS, we identified six cell types, among which the AT1 cells and basal cells were regarded as malignant epithelial cells. A single‐cell study on the development from immature to mature lungs has shown that AT1 epithelial cells can act as a unique signalling centre, secreting ligands essential for mesenchymal cell and alveolar development.^57^ Generally, PPS assumed that it is mesenchymal tissue of the bronchial wall vessels or pulmonary stroma. The findings of this study suggest that PPS is composed of some malignant epithelial cells, while concurrently demonstrating a close association between malignant AT1 cells and PPS.

In the epithelial cells of PSC, AT2 cells constituted the predominant population, and the AT2 cells of PSC gradually transition from benign to malignant, suggesting that PSC may originate from AT2 cells. The pathway enrichment results showed that malignant AT2 cells activated EMT and passivated immunorelated pathways such as INF‐γ, IL‐6_JAK_STAT3 and UV_response pathway. These pathways are highly relevant to the development, proliferation and metastasis of tumours.^58^, ^59^, ^60^ Further, unsupervised cell clustering revealed the AT2 cells could be further divided into seven clusters (Figure 4A). Ninety‐two percent of the cells within cluster 1 (AT2‐C1) were malignant (Figure 4D), and significant highly expressed HMGA2 than other clusters. HMGA2 represents a group of small chromatin‐associated proteins that act as architectural transcription factors. They directly bind to DNA sequences, consequently inducing structural alterations in the DNA molecule and modulating the transcriptional activity of target genes.^61^ The aberrant expression of HMGA2 plays a crucial role in the process of carcinogenesis, such as colorectal cancer,^61^ high‐grade serous ovarian carcinoma,^62^ NSCLC,^63^ serving as an upstream mediator for various cancer hallmarks including EMT, apoptosis and so on. The literature reported HMGA2 overexpression influence the E‐cadherin and vimentin.^64^ Thereby, the cells becoming invasive or metastatic. Moreover, HMGA2 regulates the DNA repair process by exerting control over various proteins associated with DNA repair.^65^ In our study, malignant AT2 cells showed high expression while the DNA repair pathway was inhibited, suggesting that HMGA2 may negatively regulate this pathway in PSC. Then, we further confirmed the high expression of HMGA2 in PSC through GEO data, which was also validated by the IHC. Finally, considering the pivotal role of HMGA2 in malignant tumours, we conducted a prognosis analysis for PSC based on its expression level. The results revealed that PSC patients with elevated HMGA2 expression had a poorer prognosis. At present, surgery serves as the primary treatment choice for PSC and PPS. The median survival durations and 5‐year survival percentages for surgically resected and non‐resected PPS were recorded as 39.6 months and 28.7%, respectively. For resected and non‐resected PCS, median survival times and 5‐year survival rates were 23.6 months and 31.0%, and 14.9 months and 28.2%, respectively.^8^ However, given the rarity of PSC, there are no effective molecular markers for the prognostic stratification of PSC. Our study indicates that HMGA2 could potentially serve as a potential molecular prognostic marker for PSC. Moreover, PSC is usually not sensitive to conventional chemoradiotherapy, HMGA2 supports a cancer stem cell phenotype and renders cancer cells resistant to chemotherapeutic agents.^66^, ^67^

It is worth noting that, as an important driver gene in PSC, our research has found that patients with MET mutations usually have high expression of HMGA2. Therefore, in further studies, the role of HMGA2 in providing therapeutic and prognostic guidance for PSC can be explored.

The fibroblasts of PSC and PPS were subjected to further analysis in our investigations. The malignant fibroblasts cells in PSC exhibited a higher abundance of signalling pathways associated with transmembrane receptor protein kinase activity. Malignant fibroblasts in PPS were enriched for GTPase‐related pathway. To our surprise, despite a high proportion of fibroblasts in PPS, the majority of these fibroblasts were found to be benign, while the lipofibroblasts exhibited malignancy. Some recent studies have demonstrated that lipofibroblasts in cancer‐associated fibroblasts (CAFs) also plays a crucial role in the onset and progression of tumours. In a study on the colon cancer, researchers found that KRAS mutations can activate the transcription factor TFCP2, which in turn upregulates the expression of adipogenic factors BMP4 and WNT5B, ultimately inducing CAFs to transform into a special subtype rich in lipids. These lipifibrogenesis are not inert bystanders; they actively secrete vascular endothelial growth factor A (VEGFA), directly contributing to tumour angiogenesis.^68^ A study on pancreatic cancer has shown that the loss of SETD2 can cause CAFs in the tumour to differentiate into a lipid‐rich phenotype. These CAFs provide lipids to support mitochondrial oxidative phosphorylation (OXPHOS) in the tumour through the ABCA8a transporter, thereby promoting tumour progression.^69^ This indicates that the role of lipofibroblasts in different types of cancer is highly heterogeneous, including their participation in vascular survival and metabolic regulation. A direct comparison of PSC and PPS lipofibroblasts revealed similar expression signatures between both groups, except for an enrichment in the DNA repair pathway observed in PPS lipofibroblasts. This pathway is closely associated with tumourigenesis, tumour progression and targeted immunotherapy. Future studies investigating the impact of CAFs on PPS and PSC should place greater emphasis on this pathway.

Finally, the TME of PSC and PPS was analysed by cell communication analysis. In PSC, AT2‐2 cells, which highly express HMGA2, demonstrate strong interactions with other AT2 cells and immune cell types. The ligand–receptor analysis involved strong communication probabilities mostly occur between PTPRM and CD44. According to literature reports, collagens (type I and IV) and FN1 are closely related to EMT.^70^ In PPS group, most interactions are observed among fibroblasts, CD8+T cells and macrophages. Fibroblasts play a crucial role in TME of lung cancer, interacting with various cell types.^71^, ^72^ Notably, the receptor CD44 is notably shared by all cell types in PSC and PPS, making it the most prevalent one. CD44 is an important stem cell marker.^73^ Studies have shown that HMGA2 co‐expressed or regulated stem cell marker expression to promote cancer cell growth and motility, including CD44.^74^, ^75^ The significant involvement of HMGA2 in the epithelial–mesenchymal transformation of PSC and PPS, driving tumour stemness, as well as its crucial role in chemotherapy resistance, necessitates further investigation into the underlying mechanism of action.

In this study, we revealed that PSC epithelial cells have a unique AT2 cell subtype and found that HMGA2 plays an important role in PSC epithelial–mesenchymal transformation.

In PPS, the composition of it includes a few of malignant epithelial cells, while simultaneously indicating a strong correlation between malignant AT1 cells and PPS. Fibroblasts were the majority in PPS, only lipofibroblasts were malignant. Moreover, similar expression signatures in lipofibroblasts between PSC and PPS. TME first showed the receptor CD44, a biomarker of cell stem, is prominently shared by the majority of cell types in both PSC and PPS.

However, our research also has some limitations. Firstly, there is an imbalance in sample size; we included 20 cases of PSC, but only seven cases of PPS. Secondly, there is a lack of more basic experiments for verification, due to the limited availability of tissue samples. For instance, we utilised bulk RNA sequencing data from publicly available databases to validate the elevated mRNA expression of HMGA2 in tumour tissues. In addition, cell communication analysis indicates that CD44 plays a significant role in various cell types. However, due to sample issues, we were unable to conduct characterisation by using tissue sample. In the future, we will recollect clinical samples. On one hand, we will conduct further characterisation and validation of CD44 and HMGA2. On the other hand, we will employ spatial transcriptomics to further explore the distribution patterns of different cell types in PSC and PPS, along with their more precise interactions. We will also delve deeper into the origin and development patterns of these highly heterogeneous tumours.

In conclusion, our snRNA sequencing analysis reveals distinct cellular origins and TMEs in PPS and PSC. We demonstrate that PSC is predominantly composed of malignant epithelial cells, with AT2 cells identified as a likely cellular origin undergoing malignant transformation via EMT, driven in part by the oncogenic factor HMGA2. In contrast, PPS is characterised by a majority of fibroblasts, among which only lipofibroblasts display malignant features, and also harbours a minor population of malignant epithelial cells, including AT1 cells. The TME in both malignancies is marked by complex cell–cell communication, with the stemness‐associated receptor CD44 being a central node. These findings provide critical insights into the pathogenesis of PSC and PPS at single‐cell resolution, offering a molecular basis for improving diagnostic accuracy and developing targeted therapeutic strategies for these aggressive diseases.

## AUTHOR CONTRIBUTIONS

All authors have read and approved the manuscript. **Data curation**: Jianfei Zhu, Yanlu Xiong and Tao Jiang. **Investigation**: Jun Wei and Jie Lei. **Methodology**: Shouzheng Ma, Qianqian Duan, Qin Zhang and Wanglong Deng. **Project administration**: Jie Lei and Dongsheng Chen. **Supervision**: Jiakuan Chen and Qianqian Duan. **Writing – original draft**: Wenchen Wang, Qianqian Duan, Qin Zhang and Jianfei Zhu. **Writing– review & editing**: Tao Jiang and Jie Lei.

## CONFLICT OF INTEREST STATEMENT

The authors declare no conflicts of interest.

## FUNDING INFORMATION

No.

## ETHICS STATEMENT

This human study was conducted under the approval of the Ethics Committee of The Second Affiliated Hospital, Air Force Medical University (TDLL‐202204‐01), as per the Helsinki Declaration of 1975, which was revised in 1983. Written informed consent was obtained from the patient and the relative of the patient.

## CONSENT FOR PUBLICATION

Not applicable.

## CODE AVAILABILITY

No new algorithms were developed for this study. All codes generated for analysis are available.

## Data Availability

The original contributions presented in the study are included in the article/supporting information, further inquiries can be directed to the corresponding author. Bulk‐RNA‐seq data of PSC are available from NCBI Gene Expression Omnibus under accession no. GSE110205.
